# Genomic Epidemiology and Phenotypic Characterization of Staphylococcus aureus from a Tertiary Hospital in Tianjin Municipality, Northern China

**DOI:** 10.1128/spectrum.04209-22

**Published:** 2023-02-14

**Authors:** Lu Liu, Huagang Peng, Naan Zhang, Mengyang Li, Zhaozhe Chen, Weilong Shang, Zhen Hu, Yuting Wang, Yi Yang, Dongmei Wang, Qiwen Hu, Xiancai Rao

**Affiliations:** a Department of Microbiology, School of Medicine, Chongqing University, Chongqing, China; b Department of Microbiology, College of Basic Medical Sciences, Army Medical University, Chongqing, China; c Department of Clinical Laboratory, 983th hospital of PLA, Tianjin, China; University of Calgary

**Keywords:** *Staphylococcus aureus*, whole-genome sequencing, phylogenetic analysis, antimicrobial resistance gene, virulence factor gene, biofilm formation, hemolytic activity, ST398, ST59, ST5527

## Abstract

Staphylococcus aureus remains a dangerous pathogen and poses a great threat to public health worldwide. The prevalence of the S. aureus clonotype is temporally and geographically variable. The genomic and phenotypic characteristics of S. aureus isolates in Tianjin, which is among the four big municipalities in China, are unclear. In the present study, 201 nonduplicate S. aureus isolates, including 70 methicillin-resistant S. aureus (MRSA) and 131 methicillin-susceptible S. aureus (MSSA), were collected from 2015 to 2021 in a tertiary hospital in Tianjin. Whole-genome sequencing of S. aureus isolates was carried out to investigate bacterial molecular characteristics, genomic phylogeny, antimicrobial resistance (AMR) gene carriage, and virulence factor gene distribution. The antibiotic resistance profiles, hemolytic activities, and biofilm formation abilities of the S. aureus isolates were also determined. In total, 31 distinct sequence types (STs) and 68 *spa* types were identified. ST59 (15.9%, 32/201) was the predominant clonotype, followed by ST398 (14.9%, 30/201) and several other major STs (ST1, ST5, ST6, ST22, ST25, ST188, and the newly emerging ST5527). ST59 and ST5527 mainly included MRSA isolates, while ST398 and the other major STs mainly included MSSA isolates. The unique characteristics of the S. aureus isolates belonging to the major STs were determined. ST59 isolates exhibited strong hemolytic activity, and ST398 strains had high biofilm formation capacity, while ST5527 isolates presented the greatest AMR. The genomic epidemiology and phenotypic characteristics of S. aureus isolates determined in this study will help in disease control in nosocomial environments.

**IMPORTANCE** Staphylococcus aureus is an important bacterium pathogen in tertiary hospitals, which provide rich medical resources. Tianjin is one of the four municipalities in China with a population of more than 13 million. However, the epidemiology and molecular characteristics of S. aureus isolates in Tianjin are unknown. In this study, the genomic and phenotypic analyses were performed to investigate 201 S. aureus isolates collected from a tertiary hospital in Tianjin over a time span of 6 years. The refined analysis of predominant clones ST59, ST398, the newly emerging clone ST5527, as well as other major clones, will undoubtedly aid in the control and prevention of infections caused by S. aureus in tertiary hospitals.

## INTRODUCTION

Staphylococcus aureus is a versatile pathogen that can cause many types of infections, such as skin/soft tissue infections, sepsis, bacteremia, and pneumonia (1). S. aureus is also notorious for its acquisition and carriage of diverse antimicrobial resistance (AMR) (1). After acquiring staphylococcal cassette chromosome *mec* (SCC*mec*) elements or genetic mutations, methicillin-sensitive S. aureus (MSSA) evolves into methicillin-resistant S. aureus (MRSA) (2, 3). The spread of MRSA globally has imposed a high burden on health care resources (2). Fortunately, the isolation rate of MRSA from Chinese hospitals decreased substantially from 69% in 2005 to 30% in 2021 (4). However, the increased prevalence of highly virulent MSSA clones, such as sequence type 5 (ST5), ST8, ST121, and ST188, has become a new concern (5–8).

The spread of S. aureus clones is geographically and temporally variable (9–11). The health care-associated (HA) MRSA ST239 and ST5 are globally pandemic clones (11). The community-associated (CA) MRSA ST59 clone is predominant in the Asia-Pacific area, and ST80 mainly circulates in Europe, while ST8-USA300 is prevalent in America (11). For livestock-associated MRSA (LA-MRSA) clones, ST398 is predominant in Europe, while ST9 is prevalent in Asia (11). Moreover, the replacement of S. aureus clones in specific areas has also been observed frequently (12). ST239-MRSA and ST5-MRSA are among the most prevalent HA-MRSA clones in China, while ST59-MRSA is the predominant CA-MRSA clone (13). A multicenter study showed that ST239-MRSA-III-t030 was the most predominant clone in Beijing, Guangzhou, and Urumqi between 2009 and 2012, while ST239-MRSA-III-t037 is dominant in Chongqing, and ST5-MRSA-II-t002 is predominant in Shanghai (13). In a tertiary hospital in Beijing, ST239-MRSA-III-t037 has been replaced by ST239-MRSA-III-t030 since 2000 (14). The ST239-MRSA clone has been gradually replaced by ST59-MRSA and other MRSA/MSSA clones since 2008 based on surveys in single hospital and multicenters (9, 10). Between 2015 and 2017, ST59-MRSA becomes the predominant MRSA clone in hospitals (15–18). Human-adapted ST188-MSSA and ST398-MRSA clones have also been imported into hospitals in recent years (8, 19). Upon the dynamic changes of S. aureus clonotypes, the characterization of both MRSA and MSSA isolates in a certain area is crucial for the control of infectious diseases.

Several assays, such as multilocus sequencing typing (MLST), SCC*mec* typing, staphylococcal protein A gene (*spa*) typing, and pulsed-field gel electrophoresis, have been developed to figure out the clonal distribution and molecular characteristics of S. aureus isolates (20–22). Considering the limited loci analyzed, the results derived from these typing methods reveal partial molecular characteristics of S. aureus. Recently, whole-genome sequencing (WGS) has become a powerful tool for bacterial typing. WGS data facilitate the phylogenetic analysis and epidemiological investigation of diverse pathogens, such as Pseudomonas aeruginosa, Acinetobacter baumannii, and Klebsiella pneumoniae (23). Whole- and core genome MLST (wg-MLST and cg-MLST, respectively) and single-nucleotide polymorphism (SNP) variant analyses are largely congruent in the epidemiology investigation of pathogenic bacteria (24). The data from WGS analysis present well clonal predominance, clonal replacement, and clonal evolution (15–18, 25).

The genomic and phenotypic characteristics of S. aureus isolates are not available in Tianjin, a big municipality with a population of more than 13 million. In the present study, 201 S. aureus strains from a tertiary hospital in Tianjin were collected between 2015 and 2021, and WGS was performed for all the S. aureus isolates. The STs, *spa* types, and SCC*mec* types of S. aureus isolates were identified. Results showed that ST59 was the predominant clonotype, followed by ST398 and several other major STs. ST59 and ST5527 mainly included MRSA isolates, while ST398 and the other major STs mainly included MSSA isolates. Fifteen AMR and 98 virulence factor genes among the 201 isolates were characterized. Phenotypically, ST59 isolates exhibited strong hemolytic activity, while ST398 strains presented high biofilm formation capacities. The newly emerging ST5527 isolates had the greatest AMR among all the S. aureus isolates. The genomic epidemiology and phenotypic characterization of S. aureus in Tianjin will facilitate the control and treatment of staphylococcal infections in the city.

## RESULTS

### Clinical characteristics of the S. aureus isolates.

**FIG 1:**
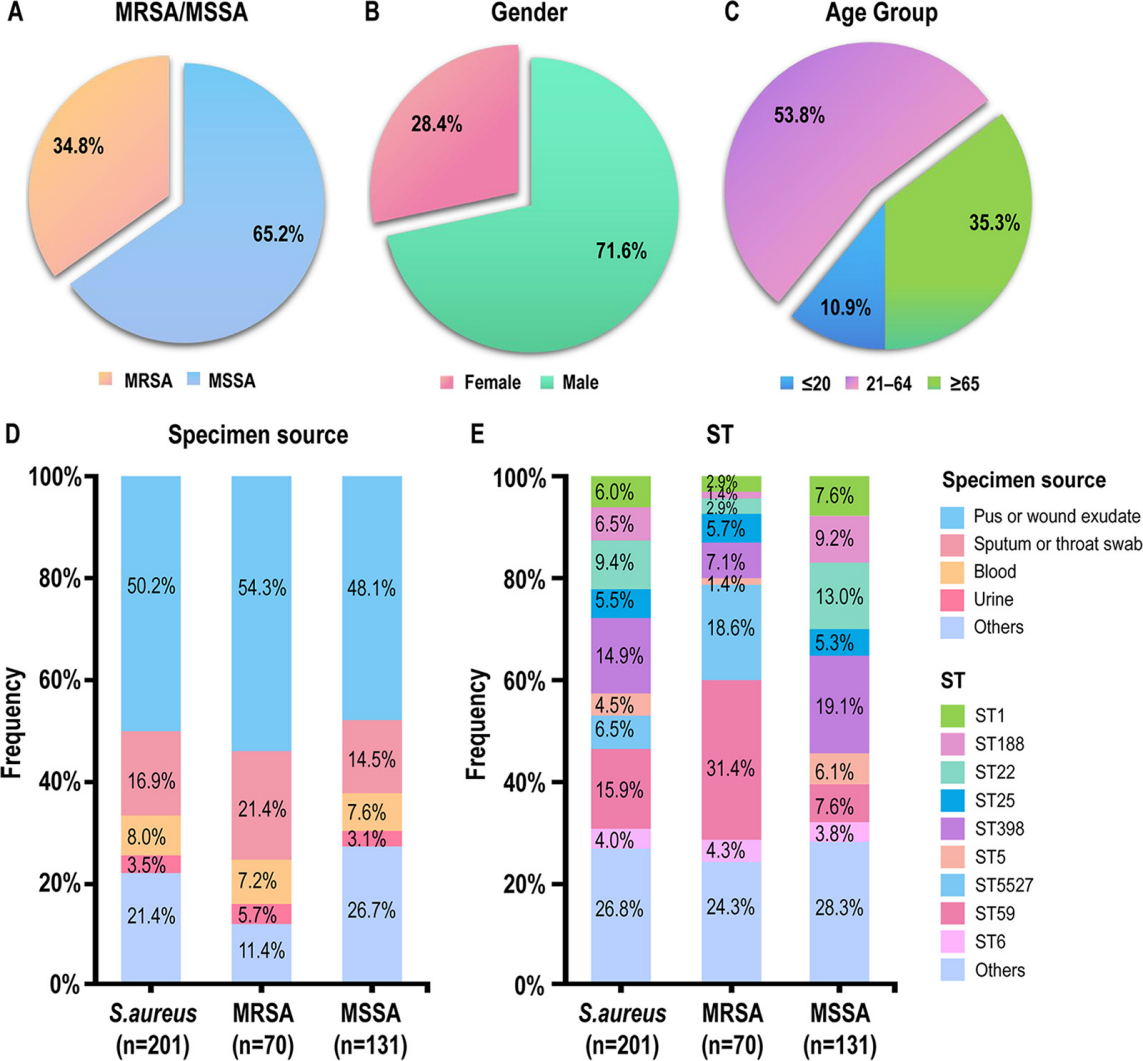
Clinical information of inpatients, specimen sources, and distribution of STs of the S. aureus strains. (A) Composition of the 201 S. aureus isolates. (B and C) Clinical information of S. aureus-infected inpatients classified by gender (B) and age (C). (D) Distribution of specimen sources in S. aureus, MRSA, and MSSA isolates. (E) Distribution of STs in S. aureus, MRSA, and MSSA strains.

In total, 201 nonduplicate clinical S. aureus isolates (70 MRSA and 131 MSSA) (Fig. 1A) were collected from inpatients in a tertiary hospital in Tianjin municipality between 2015 and 2021. As shown in Fig. 1B, male inpatients were more susceptible to S. aureus infections than female (71.6% versus 28.4%). When classified by age, more than half of the S. aureus strains (53.8%, 108/201) were obtained from inpatients aged between 21 to 64 years (Fig. 1C). Regarding the specimen source, S. aureus strains were mainly isolated from the pus or wound exudate (50.2%, 101/201), followed by sputum or throat swab (16.9%, 34/201), blood (8.0%, 16/201), urine (3.5%, 7/201), and other sterile body fluid (21.4%, 43/201) (Fig. 1D).

### MLST and *spa* types of S. aureus isolates.

In total, 31 STs were identified in 197 isolates, and the 4 remaining isolates were specified for no detectable STs. The two most frequently represented STs were ST59 (15.9%, 32/201) and ST398 (14.9%, 30/201), accounting for nearly one-third of the S. aureus isolates tested (30.8%, 62/201). The remaining STs found are shown in Fig. 1E and Table S3 in the supplemental material. Among the 31 identified STs, the characteristics of ST5527 and several other STs have not been reported. ST5527 is a single-locus variant of the ST239 clone (*yqiL*-757 in ST5527 versus *yqiL*-3 in ST239) and has been one emerging ST in Tianjin. All 31 identified STs were classified into 14 CCs, and the top 5 epidemic clonal complexes (CCs) (CC59, CC398, CC1, CC5, and CC8) occupied 68.7% (138/201) of the S. aureus isolates.

A total of 68 *spa* types were identified, with the top four prevalent *spa* types of t437 (11.4%, 23/201), t189 (6.5%, 13/201), t309 (6.5%, 13/201), and t030 (6.0%, 12/201) (Table S3). Notably, all ST188 S. aureus isolates were *spa* type t189 (100%, 13/13); 92.3% (12/13) of ST5527 strains were associated with *spa* type t030, 75.0% (8/12) of ST1 strains were *spa* type t127, 68.4% (13/19) of ST22 strains were *spa* type t309, and 59.4% (19/32) of ST59 isolates were associated with *spa* type t437. In contrast, some prevalent S. aureus STs included variable *spa* types. The S. aureus isolates of the major ST398 clonotype are composed of at least 10 *spa* types, including *spa* types t034 (26.7%, 8/30), t571 (16.7%, 5/30), t1255 (6.7%, 2/30), t011 (6.7%, 2/30), t4652 (3.3%, 1/30), t1580 (3.3%, 1/30), t1451 (3.3%, 1/30), t11729 (3.3%, 1/30), t588 (3.3%, 1/30), and nondetectable (26.7%, 8/30). ST25 S. aureus isolates included eight *spa* types (t078, t081, t349, t1102, t1701, t2992, t18617, and nondetectable). The diversified *spa* types associated with ST398 and ST25 partially revealed the genetic heterogeneity in the two S. aureus clones.

### Characteristics of MRSA and MSSA isolates.

Both MRSA (54.3%, 38/70) and MSSA (48.1%, 63/131) were mainly isolated from pus or wound exudate samples from inpatients with skin and soft tissue infections (Fig. 1D), as well as sputum and throat swab (21.4% MRSA and 14.5% MSSA). Based on the analysis of the distribution of MRSA/MSSA isolates among the major STs, ST5527 (100%, 13/13) and ST59 (68.8%, 22/32) comprised mainly of MRSA isolates, while ST188 (92.3%, 12/13), ST22 (89.5%, 17/19), ST5 (88.9%, 8/9), ST1 (83.3%, 10/12), ST398 (83.3%, 25/30), ST25 (63.3%, 7/11), and ST6 (62.5%, 5/8) comprised mainly of MSSA isolates (Table S3).

Among the 70 MRSA isolates, ST59 (31.4%, 22/70) was the most prevalent clonotype, followed by ST5527 (18.6%, 13/70), ST398 (7.1%, 5/70), and ST25 (5.7%, 4/70) (Fig. 1E; Table S3). Seven SCC*mec* types or subtypes, including SCC*mec* III[3A], IVa[2B], IVc[2B], IVg[2B], V[5C2], Vb[5C2 and 5C5], and XII[9C2], were identified in 50 MRSA isolates. Five MRSA isolates contained the *mecA* gene but lacked detection of SCC*mec* elements. The 15 remaining MRSA isolates lacked *mecA* and *mecC* genes and exhibited low levels of resistance to oxacillin (OXA) and/or cefoxitin (FOX), which may have evolved through genetic mutations as reported previously (3). SCC*mec* IV[2B] was the most common type (37.1%, 26/70), followed by SCC*mec* III[3A] (18.6%, 13/70) (Table S3). In addition, a close association was observed between STs and SCC*mec* types in which ST59-MRSA isolates were strongly associated with SCC*mec* IVa[2B] (77.3%, 17/22), and ST5527-MRSA isolates were exclusively associated with SCC*mec* III[3A] (100%, 13/13) (Table S3). Notably, one ST1-SCC*mec* IVg[2B] isolate, one ST9-SCC*mec* XII[9C2] isolate, one ST25-SCC*mec* IVa[2B] isolate, two ST6-SCC*mec* IVa[2B] isolates, and two ST72-SCC*mec* IVc[2B] isolates were sporadically identified, indicating the continuing emergence of MRSA from MSSA by acquiring the SCC*mec* elements. In comparison with MRSA isolates, the stratification of STs in MSSA isolates was more diversified. ST398 (19.1%, 25/131) was the most prevalent clonotype, followed by ST22 (13.0%, 17/131), ST188 (9.2%, 12/131), ST59 (7.6%, 10/131), and ST1 (7.6%, 10/131) (Fig. 1E; Table S3).

### Phylogenetic analysis of S. aureus isolates based on core genome SNPs.

**FIG 2:**
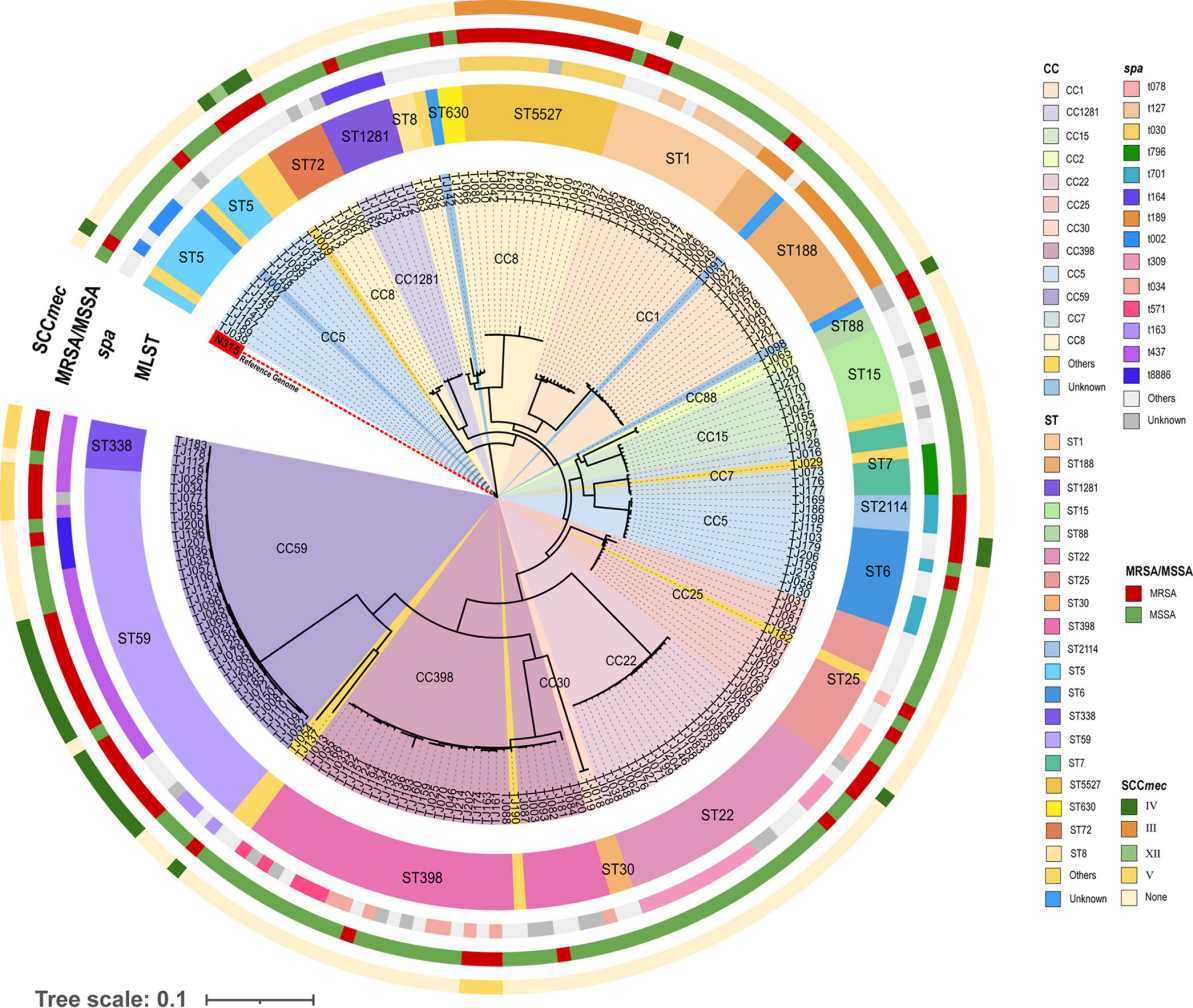
The approximate maximum-likelihood phylogenetic tree of the 201 S. aureus isolates collected in Tianjin based on 16,224 core genome SNPs. The genome sequence of N315 (a CC5-ST5 strain) was used as the reference. CCs are colored in the inner ring. Molecular characteristics of the S. aureus isolates, including STs, *spa* types, MRSA/MSSA classification, and SCC*mec* types, are colored and mapped in the outer rings.

A total of 16,224 core genome SNPs of the 201 S. aureus isolates were used to construct an approximate maximum-likelihood phylogenetic (AML) tree with fine resolution (Fig. 2). The isolates of the same ST were clustered together. Intriguingly, the isolates of ST1281 grouped together with the isolates of CC8-ST8, CC8-ST72, CC8-ST630, and CC8-ST5527, indicating that the isolates of ST1281 can also be classified into CC8 but not into CC1281. Notably, in the AML tree, eight CC5-ST6 isolates were separated from CC5-ST5 isolates. The reason behind this separation has not been determined.

### Phylogenetic reconstruction of S. aureus isolates of the major STs.

The average numbers (mean ± SD) and ranges of paired whole-genome SNPs of the isolates in major STs are summarized in Table 1. Generally, the average paired SNPs of different STs varied within the number of several hundred. However, the 13 isolates of ST5527 had a minimal number of average paired SNPs (37.4 ± 19.5), indicating the close relatedness of these isolates.

**TABLE 1:** Summary of paired whole-genome SNPs in S. aureus isolates of the major STs

ST	No. of isolates	No. of paired SNPs (mean ± SD)	Range of paired SNPs	No. of SNPs	Reference strain	GenBank accession no.
ST59	32	400.5 ± 195.7	0–691	2,735	SA40	NC_022443
ST5527	13	37.4 ± 19.5	2–74	1,312	TW20	FN433596
ST398	30	518.2 ± 132.6	4–680	4,694	71193	NC_017673
ST22	19	256.4 ± 139.6	65–773	2,025	71A_S11	CP010940
ST188	13	376.4 ± 116.4	2–605	15,212	MW2	BA000033
ST1	12	603.7 ± 202.0	5–972	2,787	MW2	BA000033
ST25	11	296.2 ± 201.6	9–730	26,221	71A_S11	CP010940
ST5	9	673.2 ± 349.1	5–997	2,227	N315	BA000018
ST6	8	384.3 ± 109.4	0–549	1,364	M2024	CP047021

**FIG 3:**
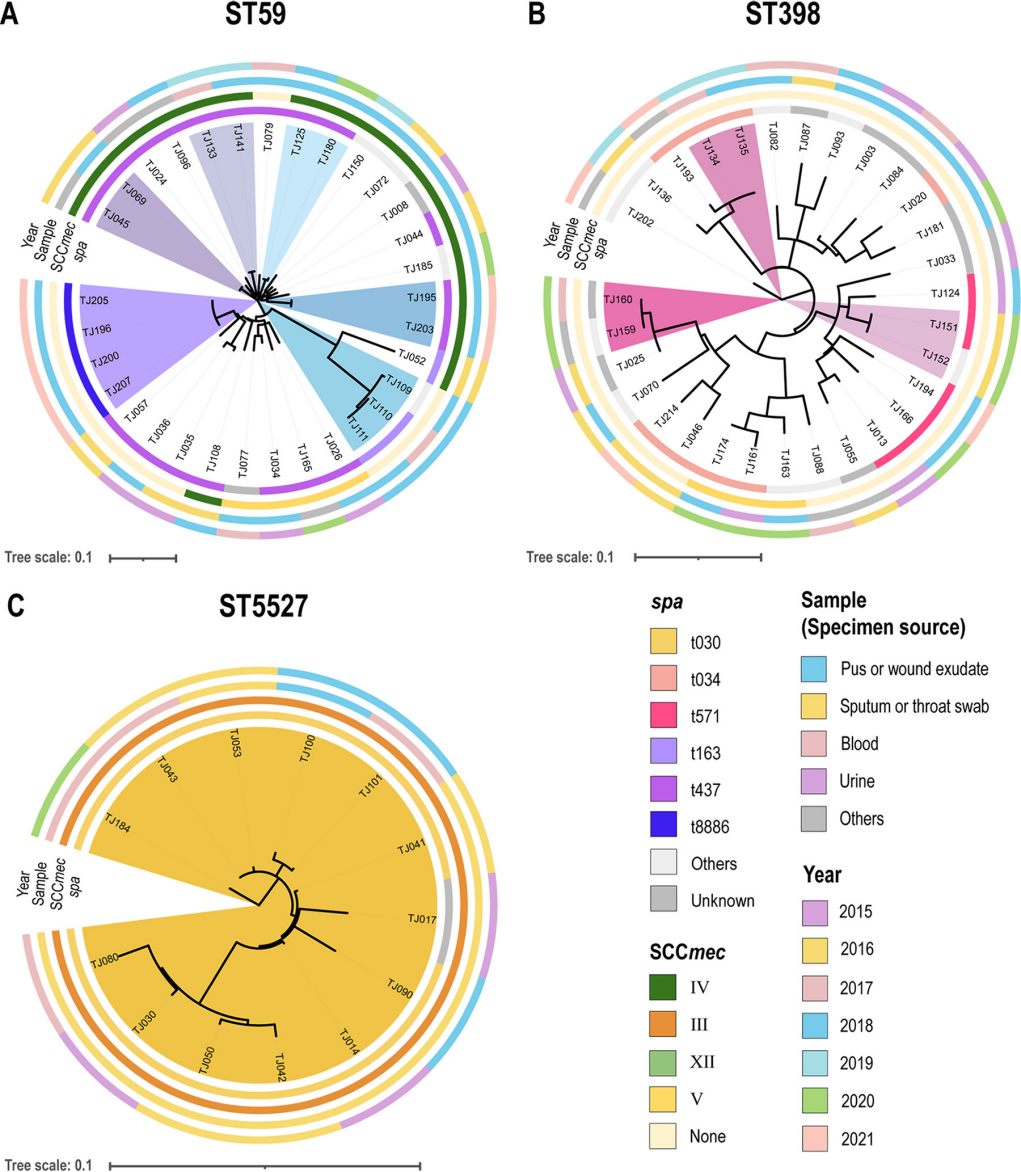
Reconstructed maximum-likelihood phylogenetic trees of S. aureus isolates belonging to ST59 (A), ST398 (B), and ST5527 (C). The *spa* types, SCC*mec* types, specimen sources, and year of isolation are colored in the outer rings. Potential transmission events are marked with different colors in the isolates (whole-genome SNPs threshold of ≤25 within 6 months).

To further reveal the potential transmission routes and possible evolutionary trajectories of S. aureus isolates in the same ST, the maximum-likelihood phylogenetic trees of the isolates belonging to the major STs were reconstructed and incorporated with time and specimen source information (Fig. 3 and Fig. S1). Notably, ST59 (68.8%, 22/32) and ST398 (40.0%, 12/30) strains were mainly isolated from pus or wound exudate samples, whereas most of the ST5527-MRSA isolates were isolated from sputum or throat swab of the inpatients with respiratory system infections (69.2%, 9/13) (Fig. 3).

The reconstructed phylogenetic trees of S. aureus isolates belonging to other STs (ST1, ST5, ST6, ST22, ST25, and ST188) are shown in Fig. S1. Coll et al. proposed a conservative cutoff of 25 whole-genome SNPs or 15 core genome SNPs, above which 95% of MRSA transmission can be figured out within 6 months (26). Based on this criterion, several potential transmission events were observed within isolates of ST59, ST398, and ST5527. Especially, all 13 ST5527 isolates were predicted to cause a 6-year lasting outbreak, which requires further investigation.

### Antimicrobial susceptibility, AMR gene carriage, and genetic point mutations in the S. aureus isolates.

**FIG 4:**
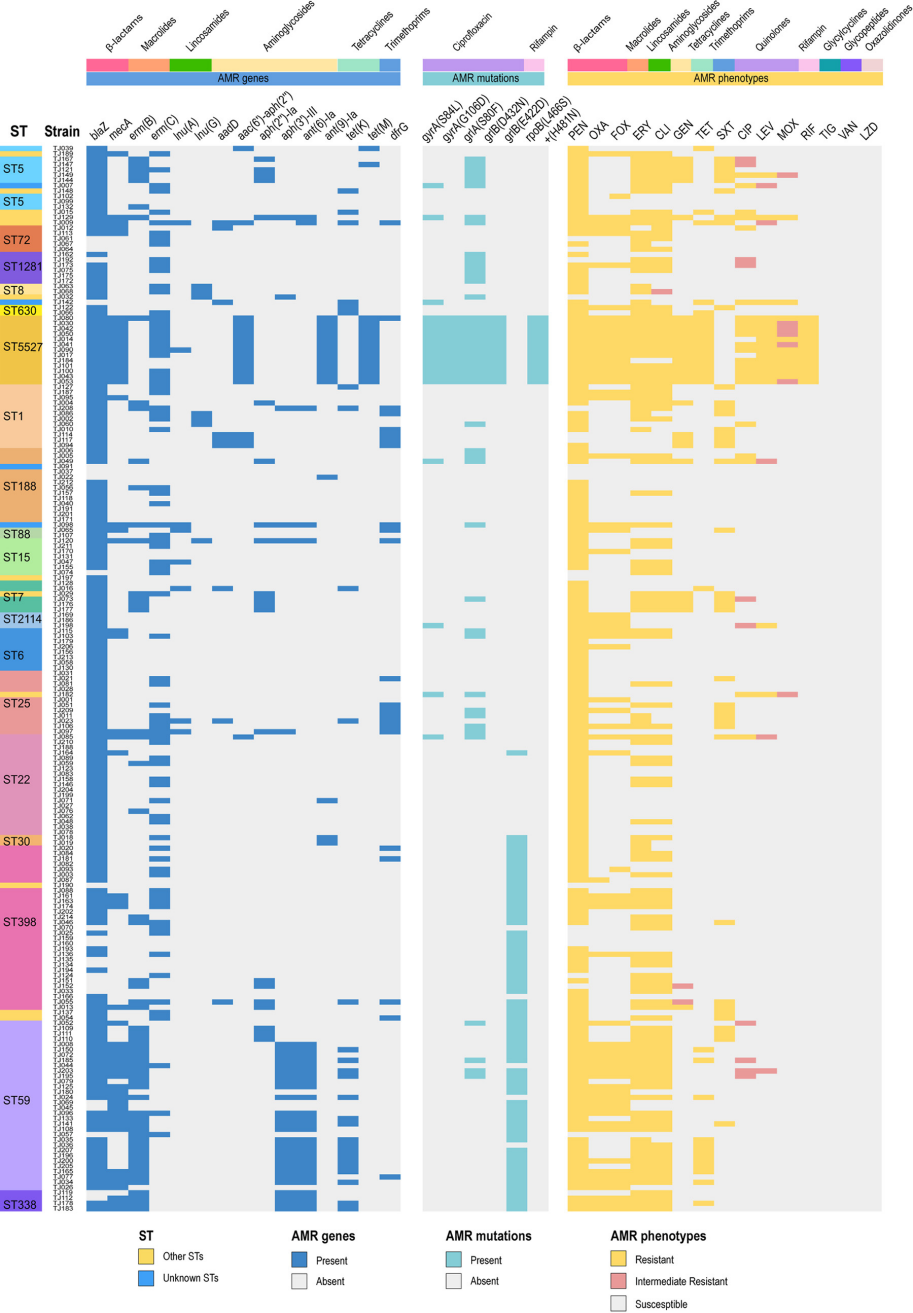
Antimicrobial resistance (AMR) gene carriages, genetic point mutations, and antibiotic susceptibility profiles of the 201 S. aureus isolates. The distributions of AMR genes, point mutations, and antibiotic susceptibility profiles are plotted against the core genome phylogeny of the S. aureus isolates. PEN, penicillin; OXA, oxacillin; FOX, cefoxitin; ERY, erythromycin; CLI, clindamycin; GEN, gentamicin; TET, tetracycline; TIG, tigecycline; CIP, ciprofloxacin; LEV, levofloxacin; MOX, moxifloxacin; SXT, trimethoprim-sulfamethoxazole; RIF, rifampin; VAN, vancomycin; LZD, linezolid.

Antimicrobial susceptibility determination revealed that all S. aureus isolates were susceptible to tigecycline (TIG), vancomycin (VAN), and linezolid (LZD) (Fig. 4 and Fig. S2). Most of the S. aureus isolates were resistant to penicillin (PEN; 89.5%, 180/201), erythromycin (ERY; 61.2%, 123/201), and clindamycin (CLI; 56.2%, 113/201). MRSA strains showed great resistance to PEN (100%, 70/70), FOX (95.7%, 67/70), OXA (92.9%, 65/70), ERY (72.9%, 51/70), and CLI (74.3%, 52/70), while MSSA isolates exhibited high-level resistance to PEN (84.0%, 110/131), ERY (55.0%, 72/131), and CLI (46.6%, 61/131). In total, 56.2% (113/201) of the S. aureus isolates were multidrug resistant (MDR) (Fig. S2). The prevalence of MDR in MRSA (75.7%, 53/70) was significantly higher than that in the MSSA isolates (45.8%, 60/131; *P *< 0.001).

AMR genes and point mutations occurred in genomes that confer resistance to quinolone and rifampin (RIF) were searched, characterized, and depicted in Fig. 4. The average AMR gene carriage by MRSA isolates (4.3 ± 2.2) was higher than those of the MSSA strains (2.1 ± 1.4, *P *< 0.05) (Fig. S3A). Macrolide resistance genes, such as *ermB* and *ermC*, exhibited different profiles in MRSA and MSSA. Aminoglycoside resistance genes like *aac(6′)-aph(2″)*, *aph(2″)-Ia*, *aph(3′)-III*, *ant(6)-Ia*, *ant(9)-Ia*, and *aadD* were highly prevalent in MRSA.

The distribution of AMR genes, point mutations, and AMR phenotypes among the S. aureus isolates of major STs is summarized in Fig. S4. Most ST59-MRSA isolates carried resistance genes of *blaZ* (90.9%, 20/22), *mecA* (95.5%, 21/22), *erm*(B) (77.3%, 17/22), *aph(3′)-III* (77.3%, 17/22), *ant(6)-Ia* (77.3%, 17/22), and *tet*(K) (45.45%, 10/22), as well as point mutation *grlB* (E422D) (90.9%, 20/22), and showed high-level resistance to PEN (100.0%, 22/22), OXA (95.5%, 21/22), FOX (95.5%, 21/22), ERY (77.3%, 17/22), and CLI (77.3%, 17/22). Most of the ST5527-MRSA isolates carried AMR genes *blaZ* (100.0%, 13/13), *mecA* (100.0%, 13/13), *erm*(C) (84.6%, 11/13), *aac(6′)*-*aph(2″)* (100.0%, 13/13), *ant*(*9*)-Ia (100.0%, 13/13), and *tet*(M) (100.0%, 13/13), as well as point mutations, such as *gyrA* (S84L+G106D; 100.0%, 13/13), *grlA* (S80F; 100.0%, 13/13), *grlB* (D432N; 100.0%, 13/13), and *rpoB* (L466S+H481N; 100.0%, 13/13). For ST59-MSSA, ST398, and other STs that mainly included MSSA isolates, most strains carried high levels of resistance genes *blaZ*, *erm*(B), or *erm*(C) and showed high-level resistance to PEN, ERY, and CLI (Fig. S4). Finally, the isolates of ST1 (50.0%, 6/12) and ST25 (63.6%, 7/11) contained the high-level AMR gene *dfrG* and exhibited high-level resistance to trimethoprim-sulfamethoxazole (SXT) (66.7% for ST1 and 54.5% for ST25).

### Virulence gene profiles of the S. aureus isolates.

**FIG 5:**
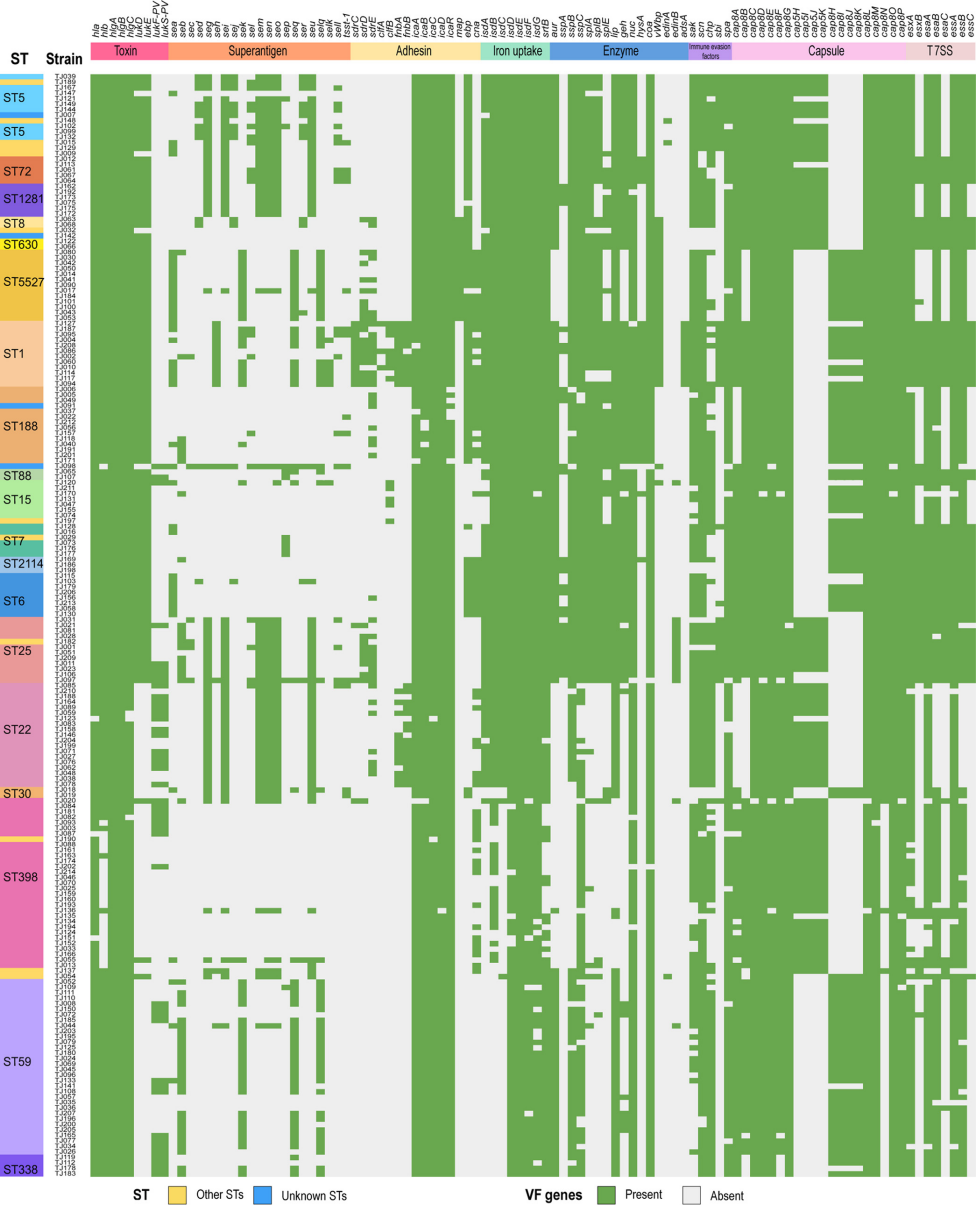
Carriage of virulence factor (VF) genes among the 201 S. aureus isolates. The distribution of VF genes is plotted against the core genome phylogeny of the S. aureus isolates. The presence of a VF gene in a strain is shown in green, and the absence of a VF gene in a strain is shown in gray.

A total of 98 virulence genes carried by S. aureus isolates were identified (Fig. 5). Most S. aureus isolates carried virulence-related genes belonging to the categories of toxin, superantigen, adhesion, iron uptake, enzyme, immune evasion, capsule synthesis, and type VII secretion system. The number of virulence genes carried by MRSA strains was comparable to that of MSSA isolates (52.5 ± 11.0 per isolate for MRSA versus 54.2 ± 8.9 per isolate for MSSA, *P* > 0.05) (Fig. S3B).

The carriage of virulence factor genes among S. aureus isolates of the major STs is summarized in Fig. S5. A strong association was found between some virulence genes and the specific STs. The *lukDE* toxin genes were totally absent in ST22 and ST59 and were only present in two isolates of ST398, although they showed a high carriage in the isolates of other STs. The *pvl* genes (*lukF-PV* and *lukS-PV*) were only present in ST22 (68.4%, 13/19), ST25 (45.5%, 5/11), ST59 (34.4%, 11/32), and ST398 (26.7%, 8/30). For superantigen genes, ST59 isolates carried characteristic enterotoxin genes *seb-sek-seq-selq* (71.9%, 23/32), ST5527 strains carried characteristic *sea-sek-seq-selq* (100%, 13/13), and ST398 isolates showed characteristic low carriage of enterotoxin genes (Fig. S5). For enzyme genes, *vWbp* was only present in the isolates of ST1 (100.0%, 12/12) and ST5527 (100.0%, 13/13), *edinB* was only present in the isolates of ST25 (100.0%, 11/11), and *adsA* was only present in ST1 (100.0%, 12/12).

S. aureus isolates of the predominant STs showed differential carriage of immune evasion genes *sak*, *scn,* and chemotaxis inhibitory protein (*chp*). In contrast, ST9 and ST630 isolates totally lacked them. For capsule types, 91.0% (183/201) of the S. aureus isolates were Cap5 (48.1%, 88/183) or Cap8 (51.9%, 95/183). Typically, ST1, ST6, ST59, ST188, and ST5527 isolates were mainly associated with the Cap8 capsule, while ST5, ST22, ST25, and ST398 isolates were associated with the Cap5 capsule. The isolates of the predominant STs carried the type VII secretion system (T7SS) but somehow exhibited differential carriage of specific T7SS components, revealing the diversity of T7SS in S. aureus.

### Biofilm formation capacity and hemolytic activity of the S. aureus isolates.

**FIG 6:**
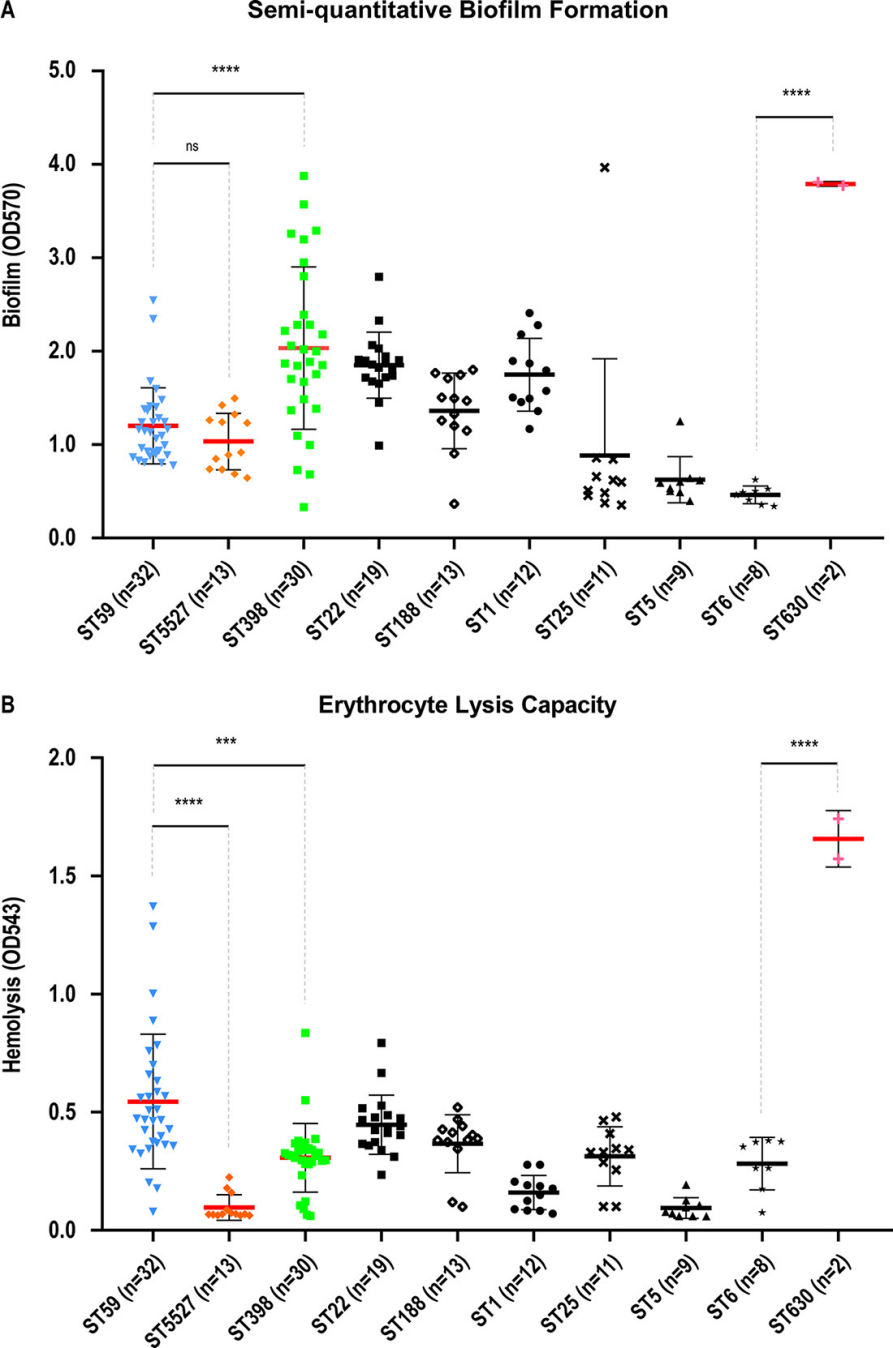
Determination of biofilm formation capacity and hemolytic activity of S. aureus isolates belonging to major STs. (A) The biofilm formation ability of the S. aureus isolates was tested by the semiquantitative crystal violet method. The absorbance measured at 570 nm of each strain is indicated. (B) Erythrocyte lysis ability of the selected S. aureus isolates of certain STs. Hemolytic activities were detected by incubating bacterial culture supernatants with rabbit red blood cells. The absorbance measured at 543 nm of each isolate is indicated. Statistical analysis was performed using one-way ANOVA. The data shown are mean ± SD of S. aureus strains in the same ST. ****, *P *< 0.0001; ***, *P *< 0.001; ns, *P *> 0.05.

Semiquantitative crystal violet assay revealed that biofilm formation varied among the S. aureus isolates of different STs (Fig. 6A). ST398 isolates exhibited different biofilm formation activities, but their average biofilm formation capacity was significantly higher than that of ST59 isolates (*P *< 0.0001). Notably, two ST630 isolates that exhibited the greatest biofilm ability were characterized (Fig. 6A).

Erythrocyte hemolysis experiments showed that S. aureus isolates of most STs exhibited comparable hemolytic capacities (Fig. 6B). However, the hemolytic activity of ST59 isolates varied and was remarkably stronger than that of the ST398 isolates. Interestingly, the isolates of ST1, ST5, and ST5527 showed reduced hemolytic activity, while the two ST630 isolates showed the strongest hemolytic ability.

## DISCUSSION

The genomic and phenotypic profiling of the S. aureus population in a certain area is important for monitoring bacterial clone prevalence and facilitating control strategy development (16). In the present study, 201 S. aureus isolates collected between 2015 and 2021 from a tertiary hospital in Tianjin were analyzed. A total of 31 STs and 68 *spa* types from the S. aureus isolates and the two most prevalent clones (ST59 and ST398) with unique antimicrobial and virulence profiles were found. ST5527-t030 is a single-locus variant of ST239-t030, which was once the most prevalent HA-MRSA clone before 2016 (10, 16). The 13 ST5527 isolates carried a large number of AMR genes and mutations in *gyrA*, *grlA*, *grlB*, and *rpoB*. This MDR nature of ST5527-t030 might confer a heavy fitness burden as is observed in ST239-t030-MRSA replacement by ST59-MRSA (17). However, ST5527-t030 isolates might have unique abilities to survive and transmit in the harsh environment of the tertiary hospital in Tianjin, similar to those of the convergent adapted two independently evolving ST239 clades in Australia (27). The narrow paired SNP distance among 13 ST5527 isolates indicated their close relatedness and suggested a potential 6-year outbreak among patients in the tertiary hospital. Accordingly, a thorough investigation of ST5527-t030 is urgently needed to fully evaluate its risk in hospital outbreaks and to formulate interventions for the control and prevention of infections caused by this newly emerging clone.

ST59 is the most successful and persistent CA-MRSA clone in Asia-Pacific areas, displaying a great overall phylogenetic diversity (28). Chen et al. identified the chemotaxis inhibitory protein (*chp*) as a strong candidate for involvement in the increased virulence potential of ST59, which might be an important driver of the MRSA lineage replacement in China (17). ST59 isolates showed high erythrocyte lytic activity, consistent with a previous report (29). The highly virulent property of the ST59 lineage might be directly related to the high incidence of ST59 infections in hospitals. In the present study, sporadic MRSA isolates of ST1, ST6, ST9, ST15, ST22, ST25, ST72, ST338 (a single-locus variant of ST59), ST398, ST630, and ST1281 were also identified. WGS-based genomic epidemiology of some of the aforementioned MRSA STs has been studied (19, 30–34). The continued emergence of new MRSA clones/isolates in hospitals further emphasizes the importance of timely surveillance.

The prevalence of MSSA among S. aureus infections increased to approximately 70% according to the CHINET reports (4). Prevalent MSSA clones such as ST1, ST7, ST15, ST22, ST59, and ST398 in hospitals have been reported, but detailed information on these STs is lacking (9, 14). ST398 is the most prevalent MSSA lineage in China, with a prevalence of 5.5% to 26.6% (35), and the prevalence of ST398-MSSA remarkably increased from 5.5% (2013 to 2014) to 18.4% (2018 to 2019) in Hainan Province (36). Human-adapted ST398-MSSA isolates contain the characteristic prophage Sa3, which usually harbors human-specific immune evasion genes *sak*, *scn*, and *chp* (35). The majority of the ST398 isolates carried *sak*, *chp*, and *scn* simultaneously. The high virulence potential and great biofilm formation ability of ST398 may jointly contribute to its prevalence in hospitals and communities. In this study, detailed information on MSSA clones such as ST1, ST5, ST6, ST22, ST25, and ST188 was also provided, portraying a full picture, when combined with other studies, of MSSA clones in Chinese hospitals (5–8).

Most MRSA isolates showed resistance to PEN, OXA, FOX, ERY, and CLI, while most of the MSSA isolates showed resistance to PEN, ERY, and CLI. The AMR profiles of specific STs provided in this study may serve as a guide for the selection of the appropriate drugs for treatment of S. aureus infections in the tertiary hospital of Tianjin with the timely assistance and application of WGS in medical practice (37). Moreover, the distributions of virulence factor genes among isolates of the major STs were searched, and some important virulence factor genes that were tightly associated with the S. aureus isolates of specific STs were found (see Fig. S4 in the supplemental material). The enterotoxin SEB of ST59 isolates in systematic infection and SAK of ST59, ST188, and ST398 isolates in the pathogenesis of S. aureus have been unveiled (38, 39). Carriage of important virulence factor genes in S. aureus isolates of specific STs may closely be related to their pathogenicity and disease progression. Information about their roles and functions in S. aureus infections may pave the way for development of antivirulence strategies (40).

This study has several limitations. First, the predominant lineages of S. aureus infections were conducted with a small sample number, which might introduce biases in the results. Multicenter studies are needed to fully evaluate S. aureus infections in China. In addition, our study used the Illumina sequencing platform, which generated short reads and gapped genomes, thus compromising our ability to reveal intact genomic profiles. The combination of next-generation sequencing techniques with third-generation sequencing platforms will generate more information and facilitate resolving the spreading of drug-resistance genes and portraying other aspects of S. aureus (23, 41). Finally, the role of the unique AMR genes and virulence factor genes associated with the predominant STs in S. aureus infections needs further experimental investigation.

In conclusion, our work uncovered that ST398 and ST59 were the two most prevalent clones in our collection of S. aureus isolates. ST59 isolates were mainly MRSA, while ST398 strains were mostly MSSA. ST59 isolates exhibited high hemolytic activity, while ST398 had high biofilm formation capacity. The characteristics of the newly emerging ST5527-MRSA and other important MSSA clones were also revealed. The data will facilitate the prevention and control of clinical S. aureus infections in the tertiary hospital of Tianjin and perhaps in other tertiary hospitals in China.

## MATERIALS AND METHODS

### Study design and collection of S. aureus isolates.

This was a laboratory-based, single-hospital retrospective study of S. aureus permitted by the Ethics Committee of Army Medical University; the requirement for informed consent was waived. Clinical data were obtained from patient electronic medical records, but patient personal information was anonymized. A total of 201 nonduplicate clinical isolates (70 MRSA and 131 MSSA isolates) from a tertiary hospital (500 beds, with admission of approximately 15,000 inpatients and 75,000 outpatients per year, Tianjin municipality, northern China) were obtained from February 2015 to May 2021 (see Table S1 in the supplemental material). S. aureus isolates were recovered from inpatients who had cough, fever, skin abscess, and other clinical symptoms related to infection. The peripheral white blood cell/neutrophil counts and/or inflammatory cytokines markers (C-reactive protein, procalcitonin, and interleukin-6) of the inpatients were elevated. Routine methods such as Gram staining, catalase assay, coagulase test, and matrix-assisted laser desorption ionization–time of flight mass spectrometry (MALDI-TOF MS) were used to further confirm the S. aureus isolates. All isolates were kept frozen at −80°C in brain heart infusion (BHI) medium supplemented with 40% glycerol.

According to the diversity of epidemic S. aureus strains, the clonal complex (CC) comprising more than 10% of the isolates was considered the major prevalent CC. A multidrug resistance (MDR) strain was determined when the isolate was resistant to at least one agent of three or more antimicrobial categories.

### Antimicrobial susceptibility testing and MRSA definition.

The antimicrobial susceptibility test was carried out using a Vitek 2 compact system and a Vitek 2 AST-GP67 test kit (bioMérieux, Inc., USA) following the instructions of the manufacturer. Fifteen antimicrobial single or combined agents were tested, including penicillin (PEN), oxacillin (OXA), cefoxitin (FOX), erythromycin (ERY), clindamycin (CLI), gentamicin (GEN), tetracycline (TET), tigecycline (TIG), ciprofloxacin (CIP), levofloxacin (LEV), moxifloxacin (MOX), trimethoprim-sulfamethoxazole (SXT), rifampin (RIF), vancomycin (VAN), and linezolid (LZD). S. aureus strain ATCC 25923 was used as the quality control, and the results were interpreted according to Clinical and Laboratory Standards Institute (CLSI) guidelines (CLSI M100-S29) (42). S. aureus isolates with OXA resistance (MIC ≥ 4 μg/mL) and/or FOX resistance (MIC ≥ 8 μg/mL) were defined as MRSA as previously described (3).

### Whole-genome sequencing, molecular typing, and phylogenetic analysis.

Each S. aureus isolate was grown in a BHI agar plate at 37°C for 16 h. Then, a single colony was picked and inoculated into fresh BHI medium and cultured at 37°C overnight. Bacterial cells were harvested by centrifugation at 12,000 × *g* at 4°C for 5 min and washed once with phosphate-buffered saline (PBS; pH 7.2). The genomic DNA of each S. aureus isolate was extracted using QIAamp DNA minikit (Qiagen, Germany) and sequenced on the Illumina NovaSeq platform (Illumina Inc., USA) using the 2 × 150-bp paired-end mode. Clean reads were obtained after removing the adapter sequences and low-quality sequences. The raw reads were trimmed and assembled into contigs using CLC Genomics Workbench software (version 21.0; CLC bio), applying the *de novo* assembly mode. The average sequencing depth was 423.96 ± 80.86, the average read count was 7.87 ± 1.06 M, and (G+C) mol% was (32.77 ± 0.28%). The average contig number of the 201 S. aureus isolates was 178.27 ± 239.31, with the contig *N*_50_ of 250.50 ± 225.34 kbp.

The assembled contigs of each isolate were used for molecular typing. STs were inferred using the MLST database (https://pubmlst.org/organisms/staphylococcus-aureus) (43). The *spa* and SCC*mec* types were determined using *spa*Typer 1.0 (https://cge.food.dtu.dk/services/spaTyper/) and SCC*mec*Finder, respectively (44, 45). The SNP variant calling method and workflow were performed. Recombination-filtered core genome SNPs generated by ParSNP were used to construct the approximate maximum-likelihood phylogenetic (AML) tree of the 201 isolates by using FastTree 2 (46, 47), and the annotation was performed with iTOL (48; https://itol.embl.de/). Moreover, the whole-genome SNPs were used to reconstruct maximum-likelihood phylogenetic trees of S. aureus isolates belonging to ST1, ST5, ST6, ST22, ST25, ST59, ST188, ST398, and ST5527 by using the general time-reversible (GTR) model of nucleotide substitution rate heterogeneity across 4 categories (GTR + Γ) in the CLC Genomics Workbench. A reference genome was used in the analyses, including read mapping, SNP calling, phylogeny tree construction, and evaluation. The GenBank accession numbers of reference genomes are provided in Table 1.

### AMR and virulence factor genes among S. aureus isolates.

The AMR and virulence factor genes of 201 S. aureus isolates were identified by CLC Genomics Workbench using the drug-resistant gene database (ResFinder database) (49) and virulence factors database (VFDB; http://www.mgc.ac.cn/VFs/) (50), respectively. The *spa* gene is ubiquitous in S. aureus. However, the retrieved *spa* carriage rate from CLC analysis was 17.9% (36/201). To reconcile these discrepancies, the results of *spa* typing (*spa*Typer 1.0) and CLC analysis were combined to probe the presence of the *spa* gene. AMR to CIP, LEV, MOX, and RIF mediated by point mutations that occurred in certain genes was identified by PointFinder (51). Distributions of AMR and virulence factor genes were managed and annotated using iTOL (48).

### Detection of virulence factor genes by PCR.

Due to the polymorphisms of some virulence factor genes, obvious discrepancies were found after analysis by CLC Workbench. PCR amplification using conserved primers for *coa*, *nuc*, *hla*, *hlb*, *hlgB*, *hlgC*, *icaC*, and *cap* genes was performed. The primers and sizes of amplicons are provided in Table S2.

### Detection of biofilm formation with semiquantitative crystal violet assay.

Semiquantitative crystal violet assay for S. aureus biofilm was performed as previously described (19). Briefly, S. aureus strains were cultured in BHI at 37°C for 16 h. Then, the culture was 1:1,000 diluted with fresh BHI medium, inoculated into 24-well plates (1 mL per well), and incubated at 37°C for 24 h. After incubation, the culture supernatants were carefully aspirated, and the bacterial cells in each well were washed once with PBS and stained with 1% (mass/vol) crystal violet (Chongqing Boer Biotech Co., China) after drying. After staining for 20 min, the residual crystal violet was washed with slow water, dried, and dissolved in 100 μL of glacial acetic acid. The optical density value at 570 nm (OD_570_) was read by micro-enzyme-linked immunosorbent assay (ELISA) autoreader (Thermo Scientific, USA).

### Erythrocyte lysis experiment.

The erythrocyte lysis experiment was carried out as described previously (52). Briefly, S. aureus isolates were grown in BHI at 37°C for 12 h, and then each culture was centrifuged at 12,000 × *g* at 4°C for 5 min, and 100 μL of the culture supernatants was collected after centrifugation. The defibered rabbit red blood cells (Guangzhou Future Biotech Co., China) were prepared with PBS (6%, vol/vol), and 100 μL of the blood cells was mixed with 100 μL of bacterial culture supernatant and incubated at 37°C for 30 min. The OD_543_ was determined. Blood cells buffered with PBS were used as negative control, while red blood cells treated with double-distilled water (ddH_2_O) were used as positive control. The ultimate percentage of hemoglobin release was calculated according to OD_543_ values.

### Statistical analysis.

Unpaired two-tailed Student's *t* test and one-way analysis of variance (ANOVA) were performed to analyze statistical significance. All data were analyzed using GraphPad Prism 8.0. *P* values of <0.05 were reported as statistically significant.

### Data availability.

The sequence data of 201 S. aureus isolates have been deposited in the Sequence Read Archive database under BioProject accession no. PRJNA876800.
