# Supplementary material for: Genomic Epidemiology and Phenotypic Characterization of Staphylococcus aureus from a Tertiary Hospital in Tianjin Municipality, Northern China
Source: Microbiol Spectr. 2023 Feb 14;11(2):e04209-22. doi: 10.1128/spectrum.04209-22 (PMC10100870; doi:10.1128/spectrum.04209-22)
Supplement: Supplemental file 1 — Supplemental material. Download spectrum.04209-22-s0001.pdf, PDF file, 3.7 MB [file spectrum.04209-22-s0001.pdf]

Supplementary Material

Five figures and three tables were provided in supplementary material.

Figure S1. Reconstructed maximum likelihood phylogenetic trees of *S. aureus* isolates belonging to ST1, ST188, ST22, ST25, ST5, and ST6.

Figure S2. Antimicrobial resistance profiles of MRSA, MSSA, MDR strains, and *S. aureus* isolates.

Figure S3. Number of certain genes carried by *S. aureus* isolates.

Figure S4. Distribution of antimicrobial resistance genes among *S. aureus* isolates of major STs.

Figure S5. Distribution of virulence factor genes among *S. aureus* isolates of major STs.

Table S1. Clinical and molecular information of the 201 *S. aureus* isolates collected from a tertiary hospital in Tianjin, Northern China.

Table S2. Primers and sizes of amplicons used for detection of virulence factor genes and capsule typing.

Table S3. Molecular characteristics of *S. aureus* isolates collected in this study.

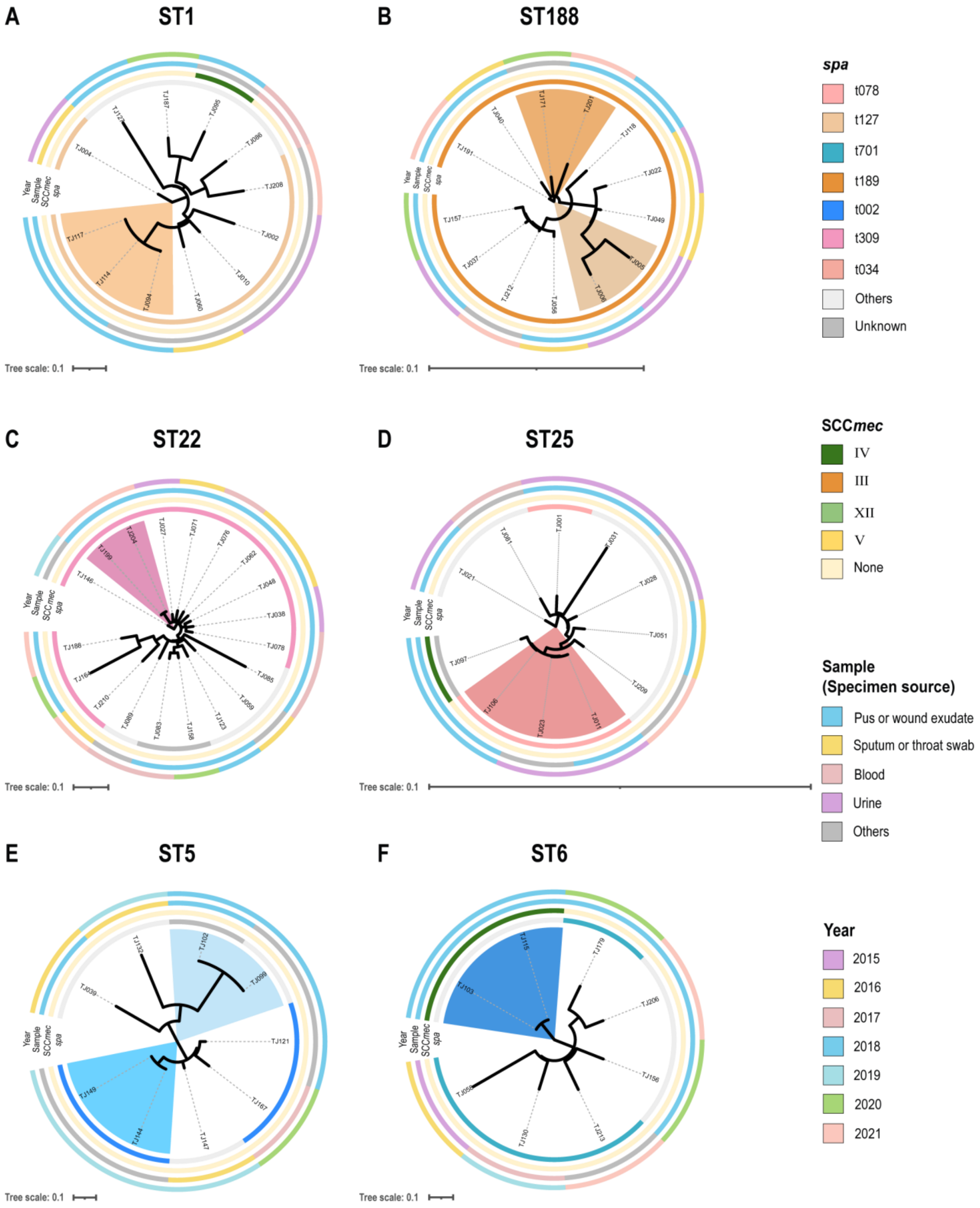

Figure S1. Reconstructed maximum likelihood (ML) phylogenetic trees of isolates belonging to (A) ST1, (B) ST188, (C) ST22, (D) ST25, (E) ST5, and (F) ST6. The *spa* types, SCC*mec* types, specimen sources, and year of isolation are colored in the outer rings. Potential transmission events are marked with different colors in the isolates (SNP threshold of ≤24).

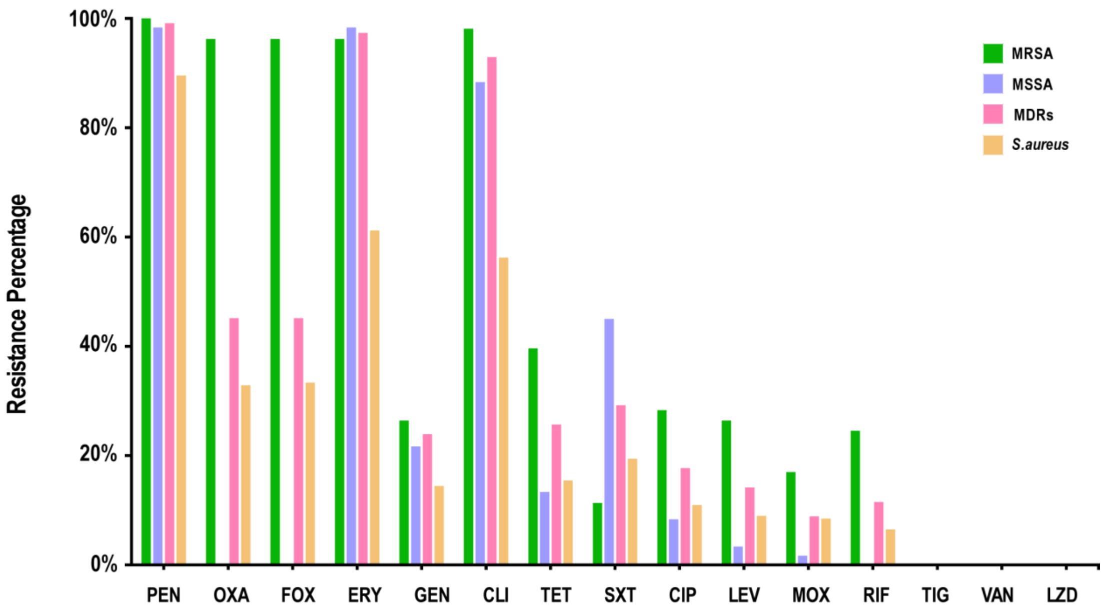

Figure S2. Antimicrobial resistance profiles of MRSA, MSSA, MDR strains, and *S. aureus* isolates. Sample antimicrobial susceptible testing of the 201 *S. aureus* isolates against a panel of 15 antimicrobial agents was determined. PEN, penicillin; OXA, oxacillin; FOX, cefoxitin; ERY, erythromycin; CLI, clindamycin; GEN, gentamicin; TET, tetracycline; TIG, tigecycline; CIP, ciprofloxacin; LEV, levofloxacin; MOX, moxifloxacin; SXT, trimethoprim/sulfamethoxazole; RIF, rifampicin; VAN, vancomycin; LZD, linezolid.

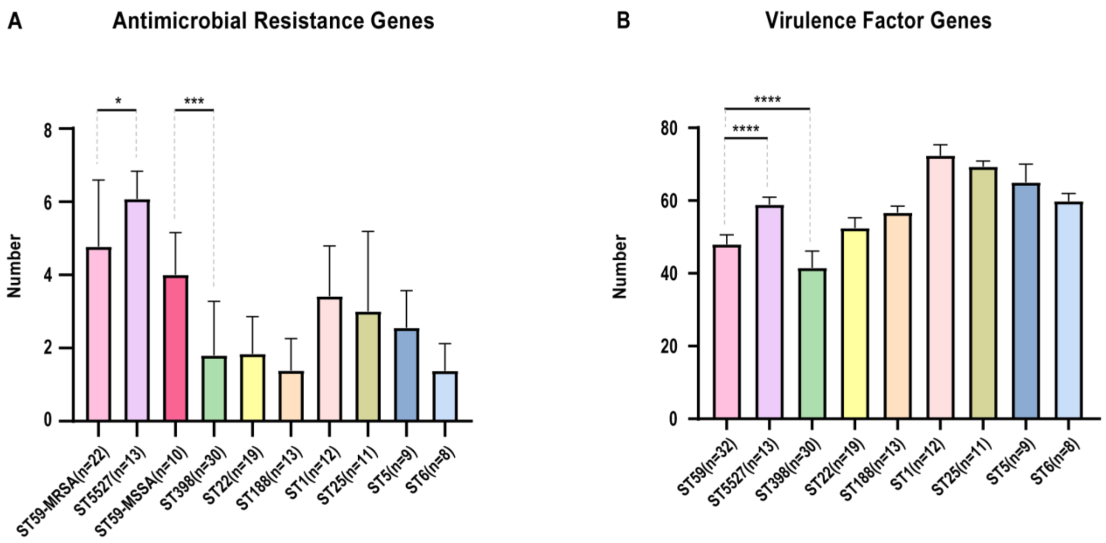

Figure S3. Number of certain genes carried by *S. aureus* isolates. (A) The antimicrobial resistance genes carried by *S. aureus* isolates of major STs. (B) The virulence factor genes carried by *S. aureus* isolates of major STs. Statistical analysis was performed using one-way ANOVA. The data shown are mean ± SD of *S. aureus* strains in the same ST. ****$P < 0.0001$, ***$P < 0.001$, and *$P < 0.05$.

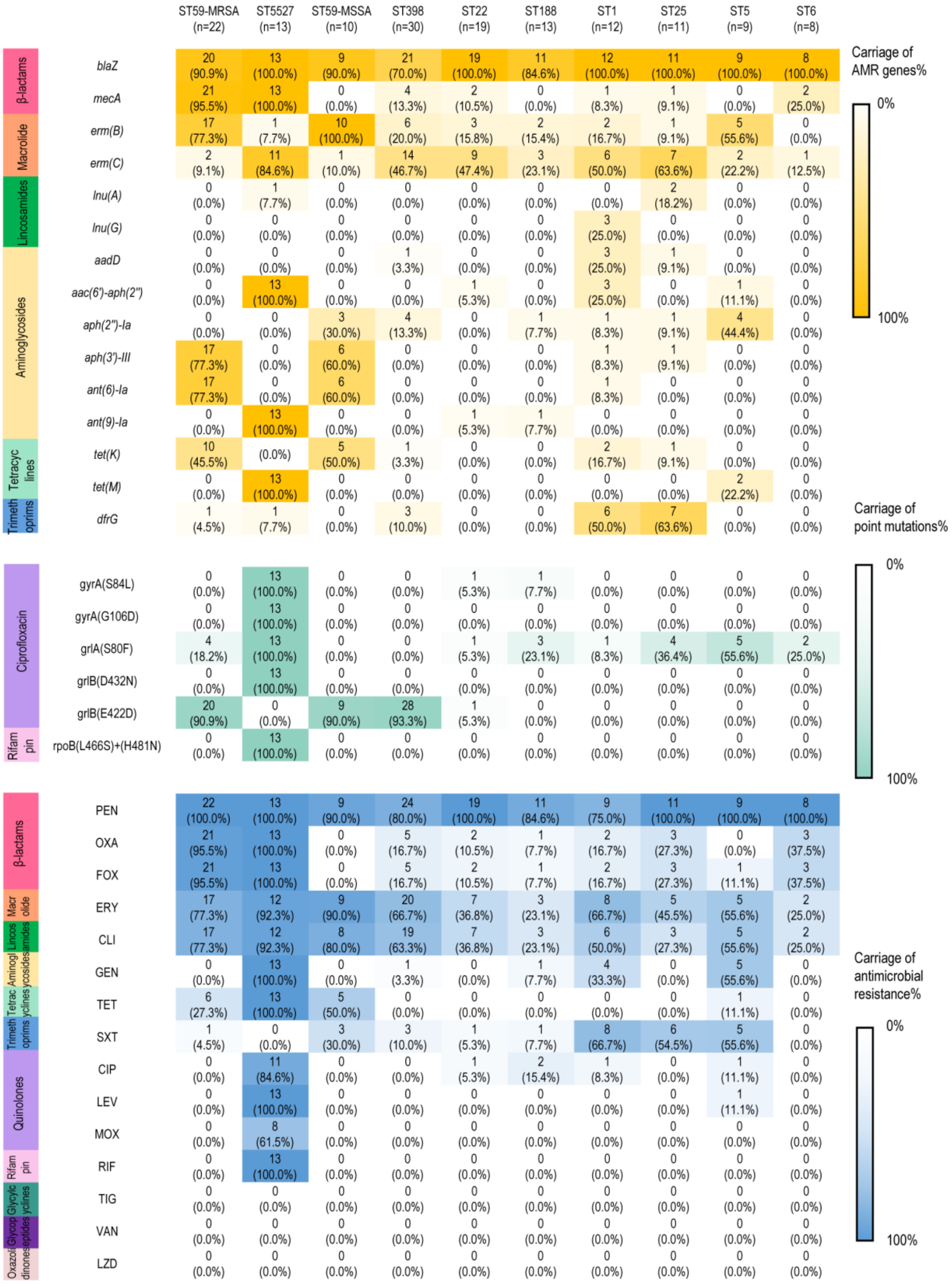

Figure S4. Distribution of antimicrobial resistance genes among *S. aureus* isolates of major sequence types

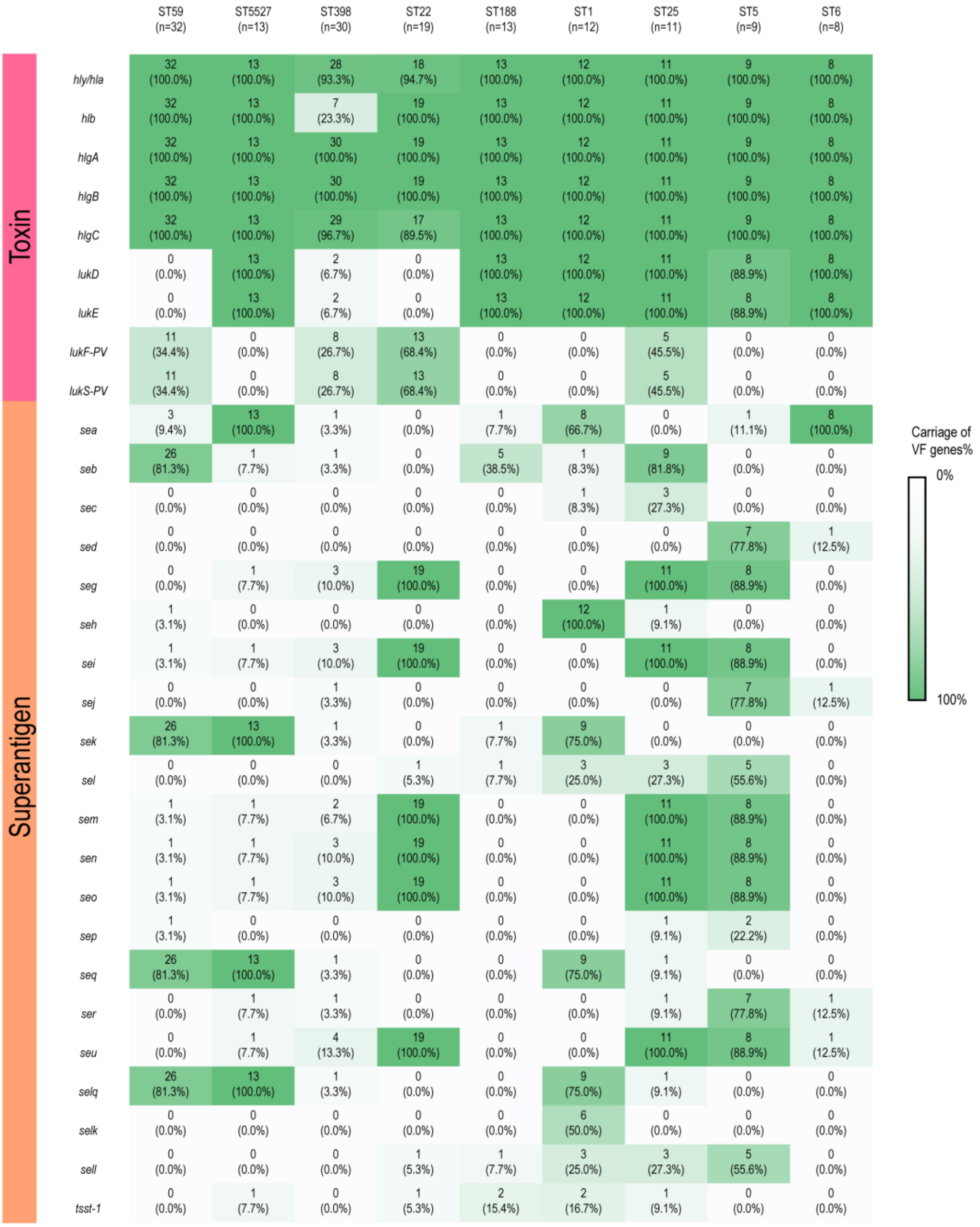
ST59 (n=32) | ST5527 (n=13) | ST398 (n=30) | ST22 (n=19) | ST188 (n=13) | ST1 (n=12) | ST25 (n=11) | ST5 (n=9) | ST6 (n=8)
Toxin
hly/hla 32 (100.0%) | 13 (100.0%) | 28 (93.3%) | 18 (94.7%) | 13 (100.0%) | 12 (100.0%) | 11 (100.0%) | 9 (100.0%) | 8 (100.0%)
hlb 32 (100.0%) | 13 (100.0%) | 7 (23.3%) | 19 (100.0%) | 13 (100.0%) | 12 (100.0%) | 11 (100.0%) | 9 (100.0%) | 8 (100.0%)
hlgA 32 (100.0%) | 13 (100.0%) | 30 (100.0%) | 19 (100.0%) | 13 (100.0%) | 12 (100.0%) | 11 (100.0%) | 9 (100.0%) | 8 (100.0%)
hlgB 32 (100.0%) | 13 (100.0%) | 30 (100.0%) | 19 (100.0%) | 13 (100.0%) | 12 (100.0%) | 11 (100.0%) | 9 (100.0%) | 8 (100.0%)
hlgC 32 (100.0%) | 13 (100.0%) | 29 (96.7%) | 17 (89.5%) | 13 (100.0%) | 12 (100.0%) | 11 (100.0%) | 9 (100.0%) | 8 (100.0%)
lukD 0 (0.0%) | 13 (100.0%) | 2 (6.7%) | 0 (0.0%) | 13 (100.0%) | 12 (100.0%) | 11 (100.0%) | 8 (88.9%) | 8 (100.0%)
lukE 0 (0.0%) | 13 (100.0%) | 2 (6.7%) | 0 (0.0%) | 13 (100.0%) | 12 (100.0%) | 11 (100.0%) | 8 (88.9%) | 8 (100.0%)
lukF-PV 11 (34.4%) | 0 (0.0%) | 8 (26.7%) | 13 (68.4%) | 0 (0.0%) | 0 (0.0%) | 5 (45.5%) | 0 (0.0%) | 0 (0.0%)
lukS-PV 11 (34.4%) | 0 (0.0%) | 8 (26.7%) | 13 (68.4%) | 0 (0.0%) | 0 (0.0%) | 5 (45.5%) | 0 (0.0%) | 0 (0.0%)
Superantigen
sea 3 (9.4%) | 13 (100.0%) | 1 (3.3%) | 0 (0.0%) | 1 (7.7%) | 8 (66.7%) | 0 (0.0%) | 1 (11.1%) | 8 (100.0%)
seb 26 (81.3%) | 1 (7.7%) | 1 (3.3%) | 0 (0.0%) | 5 (38.5%) | 1 (8.3%) | 9 (81.8%) | 0 (0.0%) | 0 (0.0%)
sec 0 (0.0%) | 0 (0.0%) | 0 (0.0%) | 0 (0.0%) | 0 (0.0%) | 1 (8.3%) | 3 (27.3%) | 0 (0.0%) | 0 (0.0%)
sed 0 (0.0%) | 0 (0.0%) | 0 (0.0%) | 0 (0.0%) | 0 (0.0%) | 0 (0.0%) | 0 (0.0%) | 7 (77.8%) | 1 (12.5%)
seg 0 (0.0%) | 1 (7.7%) | 3 (10.0%) | 19 (100.0%) | 0 (0.0%) | 0 (0.0%) | 11 (100.0%) | 8 (88.9%) | 0 (0.0%)
seh 1 (3.1%) | 0 (0.0%) | 0 (0.0%) | 0 (0.0%) | 0 (0.0%) | 12 (100.0%) | 1 (9.1%) | 0 (0.0%) | 0 (0.0%)
sei 1 (3.1%) | 1 (7.7%) | 3 (10.0%) | 19 (100.0%) | 0 (0.0%) | 0 (0.0%) | 11 (100.0%) | 8 (88.9%) | 0 (0.0%)
sej 0 (0.0%) | 0 (0.0%) | 1 (3.3%) | 0 (0.0%) | 0 (0.0%) | 0 (0.0%) | 0 (0.0%) | 7 (77.8%) | 1 (12.5%)
sek 26 (81.3%) | 13 (100.0%) | 1 (3.3%) | 0 (0.0%) | 1 (7.7%) | 9 (75.0%) | 0 (0.0%) | 0 (0.0%) | 0 (0.0%)
sel 0 (0.0%) | 0 (0.0%) | 0 (0.0%) | 1 (5.3%) | 1 (7.7%) | 3 (25.0%) | 3 (27.3%) | 5 (55.6%) | 0 (0.0%)
sem 1 (3.1%) | 1 (7.7%) | 2 (6.7%) | 19 (100.0%) | 0 (0.0%) | 0 (0.0%) | 11 (100.0%) | 8 (88.9%) | 0 (0.0%)
sen 1 (3.1%) | 1 (7.7%) | 3 (10.0%) | 19 (100.0%) | 0 (0.0%) | 0 (0.0%) | 11 (100.0%) | 8 (88.9%) | 0 (0.0%)
seo 1 (3.1%) | 1 (7.7%) | 3 (10.0%) | 19 (100.0%) | 0 (0.0%) | 0 (0.0%) | 11 (100.0%) | 8 (88.9%) | 0 (0.0%)
sep 1 (3.1%) | 0 (0.0%) | 0 (0.0%) | 0 (0.0%) | 0 (0.0%) | 0 (0.0%) | 1 (9.1%) | 2 (22.2%) | 0 (0.0%)
seq 26 (81.3%) | 13 (100.0%) | 1 (3.3%) | 0 (0.0%) | 0 (0.0%) | 9 (75.0%) | 1 (9.1%) | 0 (0.0%) | 0 (0.0%)
ser 0 (0.0%) | 1 (7.7%) | 1 (3.3%) | 0 (0.0%) | 0 (0.0%) | 0 (0.0%) | 1 (9.1%) | 7 (77.8%) | 1 (12.5%)
seu 0 (0.0%) | 1 (7.7%) | 4 (13.3%) | 19 (100.0%) | 0 (0.0%) | 0 (0.0%) | 11 (100.0%) | 8 (88.9%) | 1 (12.5%)
selq 26 (81.3%) | 13 (100.0%) | 1 (3.3%) | 0 (0.0%) | 0 (0.0%) | 9 (75.0%) | 1 (9.1%) | 0 (0.0%) | 0 (0.0%)
selk 0 (0.0%) | 0 (0.0%) | 0 (0.0%) | 0 (0.0%) | 0 (0.0%) | 6 (50.0%) | 0 (0.0%) | 0 (0.0%) | 0 (0.0%)
sell 0 (0.0%) | 0 (0.0%) | 0 (0.0%) | 1 (5.3%) | 1 (7.7%) | 3 (25.0%) | 3 (27.3%) | 5 (55.6%) | 0 (0.0%)
tsst-1 0 (0.0%) | 1 (7.7%) | 0 (0.0%) | 1 (5.3%) | 2 (15.4%) | 2 (16.7%) | 1 (9.1%) | 0 (0.0%) | 0 (0.0%)
Carriage of VF genes%
0%
100%

(Continued)

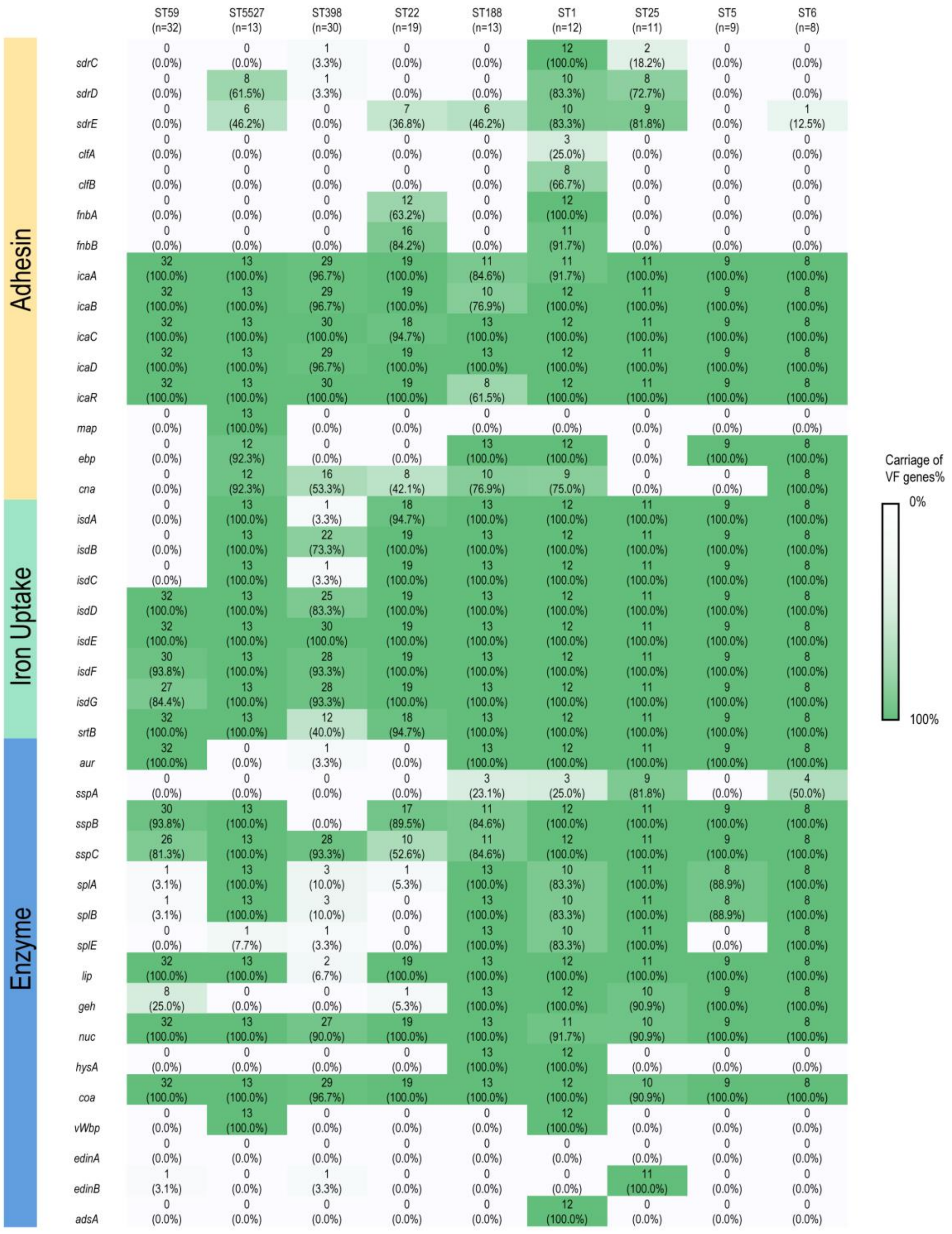
Adhesin
Iron Uptake
Enzyme
ST59 (n=32) | ST5527 (n=13) | ST398 (n=30) | ST22 (n=19) | ST188 (n=13) | ST1 (n=12) | ST25 (n=11) | ST5 (n=9) | ST6 (n=8)
sdrC 0 (0.0%) | 0 (0.0%) | 1 (3.3%) | 0 (0.0%) | 0 (0.0%) | 12 (100.0%) | 2 (18.2%) | 0 (0.0%) | 0 (0.0%)
sdrD 0 (0.0%) | 8 (61.5%) | 1 (3.3%) | 0 (0.0%) | 0 (0.0%) | 10 (83.3%) | 8 (72.7%) | 0 (0.0%) | 0 (0.0%)
sdrE 0 (0.0%) | 6 (46.2%) | 0 (0.0%) | 7 (36.8%) | 6 (46.2%) | 10 (83.3%) | 9 (81.8%) | 0 (0.0%) | 1 (12.5%)
clfA 0 (0.0%) | 0 (0.0%) | 0 (0.0%) | 0 (0.0%) | 0 (0.0%) | 3 (25.0%) | 0 (0.0%) | 0 (0.0%) | 0 (0.0%)
clfB 0 (0.0%) | 0 (0.0%) | 0 (0.0%) | 0 (0.0%) | 0 (0.0%) | 8 (66.7%) | 0 (0.0%) | 0 (0.0%) | 0 (0.0%)
fnbA 0 (0.0%) | 0 (0.0%) | 0 (0.0%) | 12 (63.2%) | 0 (0.0%) | 12 (100.0%) | 0 (0.0%) | 0 (0.0%) | 0 (0.0%)
fnbB 0 (0.0%) | 0 (0.0%) | 0 (0.0%) | 16 (84.2%) | 0 (0.0%) | 11 (91.7%) | 0 (0.0%) | 0 (0.0%) | 0 (0.0%)
icaA 32 (100.0%) | 13 (100.0%) | 29 (96.7%) | 19 (100.0%) | 11 (84.6%) | 11 (91.7%) | 11 (100.0%) | 9 (100.0%) | 8 (100.0%)
icaB 32 (100.0%) | 13 (100.0%) | 29 (96.7%) | 19 (100.0%) | 10 (76.9%) | 12 (100.0%) | 11 (100.0%) | 9 (100.0%) | 8 (100.0%)
icaC 32 (100.0%) | 13 (100.0%) | 30 (100.0%) | 18 (94.7%) | 13 (100.0%) | 12 (100.0%) | 11 (100.0%) | 9 (100.0%) | 8 (100.0%)
icaD 32 (100.0%) | 13 (100.0%) | 29 (96.7%) | 19 (100.0%) | 13 (100.0%) | 12 (100.0%) | 11 (100.0%) | 9 (100.0%) | 8 (100.0%)
icaR 32 (100.0%) | 13 (100.0%) | 30 (100.0%) | 19 (100.0%) | 8 (61.5%) | 12 (100.0%) | 11 (100.0%) | 9 (100.0%) | 8 (100.0%)
map 0 (0.0%) | 13 (100.0%) | 0 (0.0%) | 0 (0.0%) | 0 (0.0%) | 0 (0.0%) | 0 (0.0%) | 0 (0.0%) | 0 (0.0%)
ebp 0 (0.0%) | 12 (92.3%) | 0 (0.0%) | 0 (0.0%) | 13 (100.0%) | 12 (100.0%) | 0 (0.0%) | 9 (100.0%) | 8 (100.0%)
cna 0 (0.0%) | 12 (92.3%) | 16 (53.3%) | 8 (42.1%) | 10 (76.9%) | 9 (75.0%) | 0 (0.0%) | 0 (0.0%) | 8 (100.0%)
isdA 0 (0.0%) | 13 (100.0%) | 1 (3.3%) | 18 (94.7%) | 13 (100.0%) | 12 (100.0%) | 11 (100.0%) | 9 (100.0%) | 8 (100.0%)
isdB 0 (0.0%) | 13 (100.0%) | 22 (73.3%) | 19 (100.0%) | 13 (100.0%) | 12 (100.0%) | 11 (100.0%) | 9 (100.0%) | 8 (100.0%)
isdC 0 (0.0%) | 13 (100.0%) | 1 (3.3%) | 19 (100.0%) | 13 (100.0%) | 12 (100.0%) | 11 (100.0%) | 9 (100.0%) | 8 (100.0%)
isdD 32 (100.0%) | 13 (100.0%) | 25 (83.3%) | 19 (100.0%) | 13 (100.0%) | 12 (100.0%) | 11 (100.0%) | 9 (100.0%) | 8 (100.0%)
isdE 32 (100.0%) | 13 (100.0%) | 30 (100.0%) | 19 (100.0%) | 13 (100.0%) | 12 (100.0%) | 11 (100.0%) | 9 (100.0%) | 8 (100.0%)
isdF 30 (93.8%) | 13 (100.0%) | 28 (93.3%) | 19 (100.0%) | 13 (100.0%) | 12 (100.0%) | 11 (100.0%) | 9 (100.0%) | 8 (100.0%)
isdG 27 (84.4%) | 13 (100.0%) | 28 (93.3%) | 19 (100.0%) | 13 (100.0%) | 12 (100.0%) | 11 (100.0%) | 9 (100.0%) | 8 (100.0%)
srtB 32 (100.0%) | 13 (100.0%) | 12 (40.0%) | 18 (94.7%) | 13 (100.0%) | 12 (100.0%) | 11 (100.0%) | 9 (100.0%) | 8 (100.0%)
aur 32 (100.0%) | 0 (0.0%) | 1 (3.3%) | 0 (0.0%) | 13 (100.0%) | 12 (100.0%) | 11 (100.0%) | 9 (100.0%) | 8 (100.0%)
sspA 0 (0.0%) | 0 (0.0%) | 0 (0.0%) | 0 (0.0%) | 3 (23.1%) | 3 (25.0%) | 9 (81.8%) | 0 (0.0%) | 4 (50.0%)
sspB 30 (93.8%) | 13 (100.0%) | (0.0%) | 17 (89.5%) | 11 (84.6%) | 12 (100.0%) | 11 (100.0%) | 9 (100.0%) | 8 (100.0%)
sspC 26 (81.3%) | 13 (100.0%) | 28 (93.3%) | 10 (52.6%) | 11 (84.6%) | 12 (100.0%) | 11 (100.0%) | 9 (100.0%) | 8 (100.0%)
splA 1 (3.1%) | 13 (100.0%) | 3 (10.0%) | 1 (5.3%) | 13 (100.0%) | 10 (83.3%) | 11 (100.0%) | 8 (88.9%) | 8 (100.0%)
splB 1 (3.1%) | 13 (100.0%) | 3 (10.0%) | 0 (0.0%) | 13 (100.0%) | 10 (83.3%) | 11 (100.0%) | 8 (88.9%) | 8 (100.0%)
splE 0 (0.0%) | 1 (7.7%) | 1 (3.3%) | 0 (0.0%) | 13 (100.0%) | 10 (83.3%) | 11 (100.0%) | 0 (0.0%) | 8 (100.0%)
lip 32 (100.0%) | 13 (100.0%) | 2 (6.7%) | 19 (100.0%) | 13 (100.0%) | 12 (100.0%) | 11 (100.0%) | 9 (100.0%) | 8 (100.0%)
geh 8 (25.0%) | 0 (0.0%) | 0 (0.0%) | 1 (5.3%) | 13 (100.0%) | 12 (100.0%) | 10 (90.9%) | 9 (100.0%) | 8 (100.0%)
nuc 32 (100.0%) | 13 (100.0%) | 27 (90.0%) | 19 (100.0%) | 13 (100.0%) | 11 (91.7%) | 10 (90.9%) | 9 (100.0%) | 8 (100.0%)
hysA 0 (0.0%) | 0 (0.0%) | 0 (0.0%) | 0 (0.0%) | 13 (100.0%) | 12 (100.0%) | 0 (0.0%) | 0 (0.0%) | 0 (0.0%)
coa 32 (100.0%) | 13 (100.0%) | 29 (96.7%) | 19 (100.0%) | 13 (100.0%) | 12 (100.0%) | 10 (90.9%) | 9 (100.0%) | 8 (100.0%)
vWbp 0 (0.0%) | 13 (100.0%) | 0 (0.0%) | 0 (0.0%) | 0 (0.0%) | 12 (100.0%) | 0 (0.0%) | 0 (0.0%) | 0 (0.0%)
edinA 0 (0.0%) | 0 (0.0%) | 0 (0.0%) | 0 (0.0%) | 0 (0.0%) | 0 (0.0%) | 0 (0.0%) | 0 (0.0%) | 0 (0.0%)
edinB 1 (3.1%) | 0 (0.0%) | 1 (3.3%) | 0 (0.0%) | 0 (0.0%) | 0 (0.0%) | 11 (100.0%) | 0 (0.0%) | 0 (0.0%)
adsA 0 (0.0%) | 0 (0.0%) | 0 (0.0%) | 0 (0.0%) | 0 (0.0%) | 12 (100.0%) | 0 (0.0%) | 0 (0.0%) | 0 (0.0%)
Carriage of VF genes%
0%
100%

(Continued)

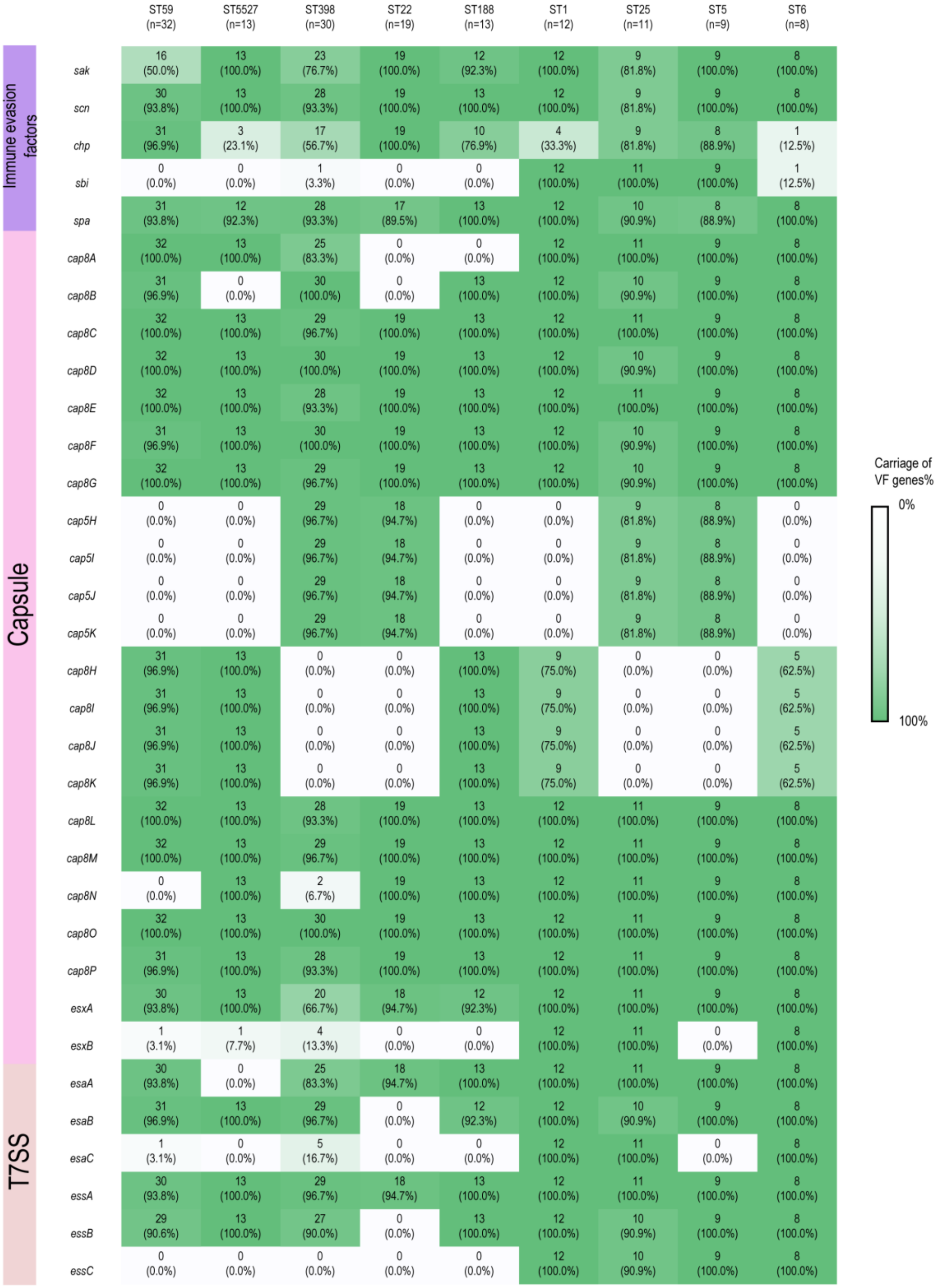

Figure S5. Distribution of virulence factor genes among *S. aureus* isolates of major STs.

Table S1. Clinical and molecular information of the 201 *S. aureus* isolates collected from a tertiary hospital in Tianjin, Northern China

Sample name	Gender	Age	Collection date	Sample type	MRSA/ MSSA	MLST type	CC	spa type	PVL	SCCmec type	Capsule type	Penicillin (PEN)	Oxacillin (OXA)	Cefoxitin(FOX)	Erythromycin (ERY)	Clindamycin (CLI)	Gentamicin (GEN)	Tetracycline (TET)	Ciprofloxacin (CIP)	Levofloxacin (LEV)	Moxifloxacin (MOX)	Trimethoprim/sulfamethoxazole (SXT)	Rifampicin (RIF)	Tigecycline (TIG)	Vancomycin (VAN)	Linezolid (LZD)
TJ001	male	36	2015/2/26	Pus or wound exudate	MRSA	ST25	CC25	t078	Absent	Absent	5	R	R	R	S	S	S	S	S	S	S	S	S	S	S	S
TJ002	female	59	2015/2/27	Others	MSSA	ST1	CC1	t127	Absent	Absent	8	R	S	S	R	R	S	S	S	S	S	S	S	S	S	S
TJ003	male	70	2015/3/13	Pus or wound exudate	MSSA	ST398	CC398	unknown	Present	Absent	5	R	S	S	R	R	S	S	S	S	S	S	S	S	S	S
TJ004	male	63	2015/3/12	Sputum or throat swab	MSSA	ST1	CC1	t127	Absent	Absent	8	R	S	S	R	R	R	S	S	S	S	R	S	S	S	S
TJ005	male	75	2015/3/17	Urine	MRSA	ST188	CC1	t189	Absent	Absent	8	R	R	R	R	R	S	S	R	S	S	S	S	S	S	S
TJ006	female	65	2015/3/17	Pus or wound exudate	MSSA	ST188	CC1	t189	Absent	Absent	8	R	S	S	S	S	S	S	S	S	S	S	S	S	S	S
TJ007	male	62	2015/3/21	Pus or wound exudate	MSSA	Unknown	Unknown	t954	Absent	Absent	5	R	S	S	R	R	S	S	R	I	S	S	S	S	S	S
TJ008	male	80	2015/4/6	Pus or wound exudate	MRSA	ST59	CC59	unknown	Present	IVa(2B)	8	R	R	R	R	R	S	S	S	S	S	S	S	S	S	S
TJ009	male	85	2015/4/6	Sputum or throat swab	MRSA	ST9	CC9	t899	Absent	XII(9C2)	5	R	R	R	R	R	S	R	S	I	S	R	S	S	S	S
TJ010	male	87	2015/4/10	Others	MSSA	ST1	CC1	t127	Absent	Absent	8	R	S	S	R	S	S	S	S	S	S	R	S	S	S	S
TJ011	male	19	2015/4/28	Pus or wound exudate	MSSA	ST25	CC25	t078	Present	Absent	5	R	S	S	R	S	S	S	S	S	S	R	S	S	S	S
TJ012	male	85	2015/5/7	Sputum or throat swab	MRSA	ST72	CC8	t664	Absent	IVc(2B)	5	R	R	R	S	R	S	S	R	S	S	S	S	S	S	S
TJ013	male	91	2015/5/19	Urine	MSSA	ST398	CC398	t571	Absent	Absent	5	R	S	S	R	R	R	S	S	S	S	R	S	S	S	S
TJ014	male	71	2015/6/21	Sputum or throat swab	MRSA	ST5527	CC8	t030	Absent	III(3A)	8	R	R	R	R	R	R	R	S	R	R	S	R	S	S	S
TJ015	male	1	2015/7/19	Pus or wound exudate	MSSA	ST3687	CC5	t045	Absent	Absent	5	R	S	S	R	R	S	R	R	S	S	S	S	S	S	S
TJ016	male	24	2015/7/21	Pus or wound exudate	MSSA	ST7	CC7	t1867	Absent	Absent	8	R	S	S	S	S	S	R	S	S	S	S	S	S	S	S
TJ017	female	75	2015/7/28	Sputum or throat swab	MRSA	ST5527	CC8	unknown	Absent	III(3A)	8	R	R	R	R	R	R	R	S	R	R	S	R	S	S	S
TJ018	male	54	2015/8/17	Others	MSSA	ST30	CC30	t021	Absent	Absent	8	R	S	S	R	R	S	S	S	S	S	S	S	S	S	S
TJ019	male	37	2015/8/19	Others	MSSA	ST30	CC30	t021	Absent	Absent	8	R	S	S	R	S	S	S	S	S	S	S	S	S	S	S
TJ020	male	20	2015/8/19	Pus or wound exudate	MSSA	ST398	CC398	t034	Present	Absent	5	R	S	S	R	S	S	S	S	S	S	S	S	S	S	S
TJ021	male	23	2015/8/21	Pus or wound exudate	MSSA	ST25	CC25	t1102	Present	Absent	5	R	S	S	S	S	S	S	S	S	S	R	S	S	S	S
TJ022	female	51	2015/9/14	Sputum or throat swab	MSSA	ST188	CC1	t189	Absent	Absent	8	S	S	S	S	S	S	S	S	S	S	S	S	S	S	S
TJ023	male	21	2015/9/18	Others	MSSA	ST25	CC25	t078	Present	Absent	5	R	S	S	R	S	S	S	S	S	S	R	S	S	S	S
TJ024	male	60	2015/9/21	Others	MRSA	ST59	CC59	t437	Absent	IVa(2B)	8	R	R	R	R	R	S	R	S	S	S	S	S	S	S	S
TJ025	female	51	2015/9/22	Sputum or throat swab	MSSA	ST398	CC398	unknown	Absent	Absent	5	S	S	S	S	S	S	S	S	S	S	S	S	S	S	S
TJ026	male	55	2015/10/1	Pus or wound exudate	MRSA	ST59	CC59	t437	Present	Vb(5C2&5)	8	R	R	R	S	S	S	S	S	S	S	S	S	S	S	S
TJ027	female	37	2015/10/8	Pus or wound exudate	MSSA	ST22	CC22	t309	Present	Absent	5	R	S	S	S	S	S	S	S	S	S	S	S	S	S	S
TJ028	male	41	2015/10/13	Others	MSSA	ST25	CC25	t2992	Absent	Absent	5	R	S	S	S	S	S	S	S	S	S	S	S	S	S	S
TJ029	female	30	2015/10/16	Others	MSSA	ST6310	Unknown	t796	Absent	Absent	8	R	S	S	R	R	R	S	S	S	S	R	S	S	S	S
TJ030	female	92	2015/11/3	Sputum or throat swab	MRSA	ST5527	CC8	t030	Absent	III(3A)	8	R	R	R	R	R	R	R	R	R	I	S	R	S	S	S
TJ031	female	52	2015/11/7	Pus or wound exudate	MSSA	ST25	CC25	t1701	Absent	Absent	5	R	S	S	S	S	S	S	S	S	S	S	S	S	S	S
TJ032	male	64	2015/11/9	Blood	MSSA	ST1821	CC8	t377	Absent	Absent	5	S	S	S	S	S	S	S	S	S	S	S	S	S	S	S
TJ033	male	26	2015/11/23	Others	MSSA	ST398	CC398	unknown	Absent	Absent	5	R	S	S	R	R	S	S	S	S	S	S	S	S	S	S
TJ034	male	50	2015/11/26	Pus or wound exudate	MRSA	ST59	CC59	t437	Present	Vb(5C2&5)	8	R	R	R	R	R	S	R	S	S	S	S	S	S	S	S
TJ035	male	89	2015/11/27	Sputum or throat swab	MSSA	ST59	CC59	t437	Absent	Absent	8	R	S	S	R	S	S	R	S	S	S	S	S	S	S	S
TJ036	female	81	2015/12/2	Pus or wound exudate	MSSA	ST59	CC59	t437	Absent	Absent	8	R	S	S	R	R	S	R	S	S	S	S	S	S	S	S
TJ037	male	58	2015/12/11	Pus or wound exudate	MSSA	ST188	CC1	t189	Absent	Absent	8	S	S	S	S	S	S	S	S	S	S	S	S	S	S	S
TJ038	male	17	2015/12/27	Pus or wound exudate	MSSA	ST22	CC22	t309	Present	Absent	5	R	S	S	S	S	S	S	S	S	S	S	S	S	S	S
TJ039	male	36	2016/1/4	Pus or wound exudate	MSSA	ST5	CC5	t688	Absent	Absent	5	R	S	S	S	S	S	R	S	S	S	S	S	S	S	S
TJ040	male	44	2016/1/10	Pus or wound exudate	MSSA	ST188	CC1	t189	Absent	Absent	8	R	S	S	S	S	S	S	S	S	S	S	S	S	S	S
TJ041	female	20	2016/1/11	Sputum or throat swab	MRSA	ST5527	CC8	t030	Absent	III(3A)	8	R	R	R	R	R	R	R	R	R	I	S	R	S	S	S
TJ042	male	86	2016/2/14	Sputum or throat swab	MRSA	ST5527	CC8	t030	Absent	III(3A)	8	R	R	R	R	R	R	R	R	R	I	S	R	S	S	S
TJ043	male	83	2016/2/18	Blood	MRSA	ST5527	CC8	t030	Absent	III(3A)	8	R	R	R	R	R	R	R	R	R	R	S	R	S	S	S
TJ044	male	19	2016/2/28	Pus or wound exudate	MRSA	ST59	CC59	t437	Absent	IVa(2B)	8	R	R	R	R	R	S	S	S	S	S	S	S	S	S	S
TJ045	male	37	2016/2/28	Others	MRSA	ST59	CC59	t437	Absent	IVa(2B)	8	R	R	R	S	S	S	S	S	S	S	S	S	S	S	S
TJ046	male	53	2016/3/26	Sputum or throat swab	MSSA	ST398	CC398	t034	Absent	Absent	5	R	S	S	R	R	S	S	S	S	S	R	S	S	S	S
TJ047	male	59	2016/3/26	Pus or wound exudate	MSSA	ST15	CC15	t084	Absent	Absent	8	R	S	S	R	R	S	S	S	S	S	S	S	S	S	S
TJ048	male	19	2016/4/5	Pus or wound exudate	MSSA	ST22	CC22	t309	Present	Absent	5	R	S	S	R	R	S	S	S	S	S	S	S	S	S	S
TJ049	female	53	2016/4/5	Sputum or throat swab	MSSA	ST188	CC1	t189	Absent	Absent	8	R	S	S	R	R	R	S	R	I	S	R	S	S	S	S
TJ050	male	86	2016/4/7	Sputum or throat swab	MRSA	ST5527	CC8	t030	Absent	III(3A)	8	R	R	R	R	R	R	R	R	R	I	S	R	S	S	S
TJ051	male	40	2016/4/13	Pus or wound exudate	MSSA	ST25	CC25	t081	Absent	Absent	5	R	S	S	R	R	S	S	S	S	S	R	S	S	S	S
TJ052	female	49	2016/4/13	Sputum or throat swab	MRSA	ST59	CC59	t163	Absent	IVa(2B)	8	R	R	R	S	S	S	S	I	S	S	S	S	S	S	S
TJ053	male	71	2016/4/25	Sputum or throat swab	MRSA	ST5527	CC8	t030	Absent	III(3A)	8	R	R	R	R	R	R	R	R	R	I	S	R	S	S	S
TJ054	male	88	2016/5/1	Sputum or throat swab	MSSA	ST121	CC121	t2019	Absent	Absent	8	R	S	S	R	R	S	S	S	S	S	R	S	S	S	S
TJ055	male	61	2016/5/5	Others	MSSA	ST398	CC398	unknown	Present	Absent	5	R	S	S	R	R	I	S	S	S	S	R	S	S	S	S
TJ056	male	63	2016/5/5	Pus or wound exudate	MSSA	ST188	CC1	t189	Absent	Absent	8	R	S	S	S	S	S	S	S	S	S	S	S	S	S	S
TJ057	male	87	2016/5/13	Sputum or throat swab	MSSA	ST59	CC59	t437	Absent	Absent	8	S	S	S	S	S	S	S	S	S	S	S	S	S	S	S
TJ058	female	86	2016/5/13	Urine	MSSA	ST6	CC5	t701	Absent	Absent	8	R	S	S	S	S	S	S	S	S	S	S	S	S	S	S
TJ059	male	9	2016/5/21	Others	MSSA	ST22	CC22	t15234	Present	Absent	5	R	S	S	R	R	S	S	S	S	S	S	S	S	S	S
TJ060	male	62	2016/5/22	Others	MSSA	ST1	CC1	t127	Absent	Absent	unknown	R	S	S	S	S	S	S	R	S	S	S	S	S	S	S
TJ061	female	65	2016/8/5	Pus or wound exudate	MSSA	ST72	CC8	t148	Absent	Absent	5	S	S	S	R	R	S	S	S	S	S	S	S	S	S	S
TJ062	male	85	2016/8/5	Pus or wound exudate	MSSA	ST22	CC22	t309	Present	Absent	5	R	S	S	S	S	S	S	S	S	S	S	S	S	S	S
TJ063	male	28	2016/8/16	Pus or wound exudate	MSSA	ST8	CC8	t9101	Absent	Absent	5	R	S	S	R	S	S	S	S	S	S	S	S	S	S	S
TJ064	female	65	2016/8/30	Pus or wound exudate	MSSA	ST72	CC8	t148	Present	Absent	5	S	S	S	R	R	S	S	S	S	S	S	S	S	S	S
TJ065	male	46	2016/9/4	Pus or wound exudate	MRSA	ST88	CC88	unknown	Absent	Absent	8	R	R	R	S	S	S	S	S	S	S	R	S	S	S	S
TJ066	male	39	2016/9/9	Blood	MSSA	ST630	CC8	t11041	Absent	Absent	5	R	S	S	R	R	S	R	S	S	S	S	S	S	S	S
TJ067	male	28	2016/9/18	Pus or wound exudate	MSSA	ST72	CC8	unknown	Absent	Absent	5	R	S	S	R	S	S	S	S	S	S	S	S	S	S	S
TJ068	male	28	2016/9/28	Pus or wound exudate	MSSA	ST8	CC8	t9101	Absent	Absent	5	R	S	S	R	I	S	S	S	S	S	S	S	S	S	S
TJ069	male	38	2016/10/10	Pus or wound exudate	MRSA	ST59	CC59	t437	Absent	IVa(2B)	8	R	R	R	S	S	S	S	S	S	S	S	S	S	S	S
TJ070	male	65	2016/10/10	Pus or wound exudate	MSSA	ST398	CC398	t588	Absent	Absent	5	S	S	S	R	R	S	S	S	S	S	S	S	S	S	S
TJ071	male	22	2016/11/12	Pus or wound exudate	MSSA	ST22	CC22	t309	Present	Absent	5	R	S	S	S	S	S	S	S	S	S	S	S	S	S	S
TJ072	male	20	2016/11/12	Pus or wound exudate	MRSA	ST59	CC59	t441	Present	IVa(2B)	8	R	R	R	R	R	S	S	S	S	S	S	S	S	S	S
TJ073	female	58	2017/1/24	Pus or wound exudate	MSSA	ST7	CC7	t796	Absent	Absent	8	R	S	S	R	R	R	S	I	S	S	R	S	S	S	S
TJ074	male	19	2017/4/22	Sputum or throat swab	MSSA	ST15	CC15	t11579	Absent	Absent	unknown	S	S	S	R	R	S	S	S	S	S	S	S	S	S	S
TJ075	male	73	2017/4/26	Others	MSSA	ST1281	CC1281	t164	Absent	Absent	5	R	S	S	R	R	S	S	S	S	S	S	S	S	S	S
TJ076	male	19	2017/5/22	Pus or wound exudate	MSSA	ST22	CC22	t309	Present	Absent	5	R	S	S	S	S	S	S	S	S	S	S	S	S	S	S
TJ077	female	61	2017/6/9	Pus or wound exudate	MRSA	ST59	CC59	unknown	Present	Vb(5C2&5)	8	R	R	R	R	R	S	S	S	S	S	S	S	S	S	S
TJ078	male	20	2017/6/14	Pus or wound exudate	MSSA	ST22	CC22	t309	Present	Absent	5	R	S	S	S	S	S	S	S	S	S	S	S	S	S	S
TJ079	male	23	2017/6/12	Pus or wound exudate	MSSA	ST59	CC59	t437	Absent	Absent	8	R	S	S	R	R	S	S	S	S	S	S	S	S	S	S
TJ080	female	93	2017/6/17	Sputum or throat swab	MRSA	ST5527	CC8	t030	Absent	III(3A)	8	R	R	R	R	R	R	R	R	R	R	S	R	S	S	S
TJ081	female	61	2017/6/25	Others	MSSA	ST25	CC25	t18617	Absent	Absent	5	R	S	S	R	R	S	S	S	S	S	S	S	S	S	S
TJ082	male	19	2017/8/16	Pus or wound exudate	MSSA	ST398	CC398	t1255	Present	Absent	5	R	S	S	S	S	S	S	S	S	S	S	S	S	S	S
TJ083	male	19	2017/9/1	Pus or wound exudate	MSSA	ST22	CC22	unknown	Present	Absent	5	R	S	S	S	S	S	S	S	S	S	S	S	S	S	S
TJ084	male	77	2017/10/6	Pus or wound exudate	MSSA	ST398	CC398	unknown	Present	Absent	5	R	S	S	R	R	S	S	S	S	S	S	S	S	S	S
TJ085	male	19	2017/10/9	Pus or wound exudate	MRSA	ST22	CC22	t005	Present	Absent	5	R	R	R	R	R	S	S	R	I	S	R	S	S	S	S
TJ086	female	55	2017/10/12	Blood	MSSA	ST1	CC1	t948	Absent	Absent	8	R	S	S	R	S	S	S	S	S	S	R	S	S	S	S
TJ087	male	70	2017/10/22	Sputum or throat swab	MSSA	ST398	CC398	unknown	Present	Absent	5	R	R	S	S	S	S	S	S	S	S	S	S	S	S	S
TJ088	male	52	2017/11/23	Others	MSSA	ST398	CC398	t4652	Absent	Absent	5	R	S	S	R	R	S	S	S	S	S	S	S	S	S	S
TJ089	female	67	2017/12/22	Others	MSSA	ST22	CC22	t3668	Present	Absent	5	R	S	S	R	R	S	S	S	S	S	S	S	S	S	S
TJ090	male	78	2018/1/18	Sputum or throat swab	MRSA	ST5527	CC8	t030	Absent	III(3A)	8	R	R	R	R	R	R	R	R	R	R	S	R	S	S	S
TJ091	female	78	2018/1/24	Others	MSSA	Unknown	Unknown	t2883	Absent	Absent	8	S	S	S	S	S	S	S	S	S	S	S	S	S	S	S
TJ093	male	20	2018/2/27	Pus or wound exudate	MRSA	ST398	CC398	t11729	Present	Absent	5	R	S	R	S	S	S	S	S	S	S	S	S	S	S	S
TJ094	male	33	2018/3/1	Others	MSSA	ST1	CC1	t127	Absent	Absent	8	R	S	S	S	S	R	S	S	S	S	R	S	S	S	S
TJ095	male	36	2018/3/25	Others	MRSA	ST1	CC1	t114	Absent	IVg(2B)	8	R	R	R	R	R	S	S	S	S	S	S	S	S	S	S
TJ096	male	23	2018/3/26	Others	MRSA	ST59	CC59	t437	Absent	IVa(2B)	8	R	R	R	R	R	S	S	S	S	S	S	S	S	S	S
TJ097	male	37	2018/3/28	Pus or wound exudate	MRSA	ST25	CC25	unknown	Present	IVa(2B)	unknown	R	S	S	S	S	S	S	S	S	S	S	S	S	S	S
TJ098	male	17	2018/4/1	Pus or wound exudate	MRSA	Unknown	Unknown	unknown	Present	IVa(2B)	unknown	R	R	R	R	R	S	S	S	S	S	S	S	S	S	S
TJ099	male	45	2018/4/16	Others	MSSA	ST5	CC5	t5353	Absent	Absent	5	R	S	S	S	S	S	S	S	S	S	S	S	S	S	S
TJ100	male	88	2018/4/21	Pus or wound exudate	MRSA	ST5527	CC8	t030	Absent	III(3A)	8	R	R	R	R	R	R	R	R	R	R	S	R	S	S	S
TJ101	male	88	2018/4/30	Blood	MRSA	ST5527	CC8	t030	Absent	III(3A)	8	R	R	R	R	R	R	R	R	R	R	S	R	S	S	S
TJ102	male	45	2018/5/4	Pus or wound exudate	MRSA	ST5	CC5	unknown	Absent	Absent	5	R	S	R	S	S	S	S	S	S	S	S	S	S	S	S
TJ103	female	50	2018/5/21	Pus or wound exudate	MRSA	ST6	CC5	t304	Absent	IVa(2B)	unknown	R	R	R	R	R	S	S	S	S	S	S	S	S	S	S
TJ106	female	69	2018/6/20	Pus or wound exudate	MRSA	ST25	CC25	t078	Present	Absent	unknown	R	R	R	R	R	S	S	S	S	S	R	S	S	S	S
TJ107	male	54	2018/6/21	Blood	MSSA	ST88	CC88	t3155	Present	Absent	8	R	S	S	S	S	S	S	S	S	S	S	S	S	S	S
TJ108	male	61	2018/6/25	Sputum or throat swab	MRSA	ST59	CC59	t437	Present	IVa(2B)	8	R	R	R	R	R	S	S	S	S	S	S	S	S	S	S
TJ109	male	76	2018/6/27	Pus or wound exudate	MSSA	ST59	CC59	t3736	Absent	Absent	8	R	S	S	R	R	S	S	S	S	S	R	S	S	S	S
TJ110	male	76	2018/6/27	Blood	MSSA	ST59	CC59	t163	Absent	Absent	8	R	S	S	R	R	S	S	S	S	S	R	S	S	S	S
TJ111	male	76	2018/7/16	Pus or wound exudate	MSSA	ST59	CC59	t163	Absent	Absent	8	R	S	S	R	R	S	S	S	S	S	R	S	S	S	S
TJ112	male	34	2018/7/31	Pus or wound exudate	MRSA	ST338	CC59	t437	Present	Vb(5C2&5)	8	R	R	R	R	R	S	S	S	S	S	S	S	S	S	S
TJ113	male	94	2018/8/11	Others	MRSA	ST72	CC8	t324	Absent	IVc(2B)	unknown	R	R	R	R	R	S	S	S	S	S	S	S	S	S	S
TJ114	male	34	2018/8/15	Pus or wound exudate	MSSA	ST1	CC1	t127	Absent	Absent	8	S	S	S	S	S	R	S	S	S	S	R	S	S	S	S
TJ115	female	50	2018/8/27	Pus or wound exudate	MRSA	ST6	CC5	t304	Absent	IVa(2B)	unknown	R	R	R	R	R	S	S	S	S	S	S	S	S	S	S
TJ117	male	34	2018/9/5	Pus or wound exudate	MSSA	ST1	CC1	t127	Absent	Absent	unknown	S	S	S	S	S	R	S	S	S	S	R	S	S	S	S
TJ118	male	20	2018/9/4	Pus or wound exudate	MSSA	ST188	CC1	t189	Absent	Absent	8	R	S	S	S	S	S	S	S	S	S	S	S	S	S	S
TJ119	male	35	2018/9/10	Pus or wound exudate	MSSA	ST338	CC59	t437	Present	Absent	8	S	S	S	R	R	S	S	S	S	S	S	S	S	S	S
TJ120	male	87	2018/9/12	Urine	MRSA	ST15	CC15	t084	Present	Absent	unknown	R	S	S	R	R	S	S	S	S	S	S	S	S	S	S
TJ121	male	55	2018/9/20	Others	MSSA	ST5	CC5	t002	Absent	Absent	unknown	R	S	S	R	R	R	S	S	S	S	R	S	S	S	S
TJ122	female	60	2018/9/25	Pus or wound exudate	MRSA	ST630	CC8	t377	Absent	Absent	5	R	R	R	R	R	S	S	S	S	S	R	S	S	S	S
TJ123	female	52	2018/10/28	Pus or wound exudate	MSSA	ST22	CC22	t16061	Present	Absent	unknown	R	S	S	S	S	S	S	S	S	S	S	S	S	S	S
TJ124	male	38	2018/12/18	Urine	MSSA	ST398	CC398	t571	Absent	Absent	5	S	S	S	R	R	S	S	S	S	S	S	S	S	S	S
TJ125	male	36	2018/12/23	Pus or wound exudate	MRSA	ST59	CC59	t437	Absent	IVa(2B)	8	R	R	R	R	R	S	S	S	S	S	S	S	S	S	S
TJ127	female	22	2018/12/27	Pus or wound exudate	MSSA	ST1	CC1	t286	Absent	Absent	unknown	R	S	S	R	R	S	S	S	S	S	R	S	S	S	S
TJ128	male	21	2019/1/23	Sputum or throat swab	MSSA	ST7	CC7	t18004	Absent	Absent	8	R	S	S	S	S	S	S	S	S	S	S	S	S	S	S
TJ129	male	51	2019/2/9	Sputum or throat swab	MRSA	ST965	CC5	t062	Absent	IVc(2B)	5	R	R	R	R	R	R	S	R	R	R	R	S	S	S	S
TJ130	female	73	2019/2/24	Blood	MSSA	ST6	CC5	t701	Absent	Absent	unknown	R	S	S	S	S	S	S	S	S	S	S	S	S	S	S
TJ131	male	74	2019/2/26	Pus or wound exudate	MSSA	ST15	CC15	t2325	Absent	Absent	8	R	S	S	S	S	S	S	S	S	S	S	S	S	S	S
TJ132	male	89	2019/3/21	Sputum or throat swab	MSSA	ST5	CC5	t2958	Absent	Absent	5	R	S	S	S	S	S	S	S	S	S	S	S	S	S	S
TJ133	female	64	2019/4/13	Blood	MRSA	ST59	CC59	t437	Present	IVa(2B)	8	R	S	S	R	R	S	S	S	S	S	S	S	S	S	S
TJ134	female	64	2019/4/11	Blood	MSSA	ST398	CC398	t034	Absent	Absent	5	R	S	S	R	R	S	S	S	S	S	S	S	S	S	S
TJ135	male	44	2019/5/1	Pus or wound exudate	MSSA	ST398	CC398	t034	Absent	Absent	unknown	R	S	S	R	R	S	S	S	S	S	S	S	S	S	S
TJ136	male	89	2019/5/1	Sputum or throat swab	MRSA	ST398	CC398	t1255	Absent	Absent	5	R	R	R	R	R	S	S	S	S	S	S	S	S	S	S

TJ137	male	55	2019/5/4	Others	MSSA	ST944	Unknown	t616	Absent	Absent	5	R	S	S	S	S	S	S	S	S	S	R	S	S	S	S
TJ141	female	38	2019/5/19	Pus or wound exudate	MRSA	ST59	CC59	t437	Present	IVa(2B)	unknown	R	R	R	R	R	S	S	S	S	S	R	S	S	S	S
TJ142	male	57	2019/5/25	Others	MSSA	Unknown	Unknown	t377	Absent	Absent	5	R	S	S	R	S	S	R	R	R	R	S	S	S	S	S
TJ144	male	20	2019/9/14	Others	MSSA	ST5	CC5	t002	Absent	Absent	5	R	S	S	R	R	R	S	S	S	S	R	S	S	S	S
TJ146	male	47	2019/10/23	Others	MSSA	ST22	CC22	t309	Present	Absent	5	R	S	S	R	R	S	S	S	S	S	S	S	S	S	S
TJ147	female	88	2019/10/22	Sputum or throat swab	MSSA	ST5	CC5	t653	Absent	Absent	5	R	S	S	R	R	R	S	I	S	S	R	S	S	S	S
TJ148	female	33	2019/10/30	Others	MSSA	ST950	CC5	t895	Absent	Absent	unknown	R	S	S	R	R	S	S	S	S	S	R	S	S	S	S
TJ149	male	21	2019/11/10	Others	MSSA	ST5	CC5	t002	Absent	Absent	5	R	S	S	R	R	R	S	R	R	I	R	S	S	S	S
TJ150	male	52	2019/12/3	Pus or wound exudate	MRSA	ST59	CC59	t441	Present	IVa(2B)	8	R	R	R	R	R	S	R	S	S	S	S	S	S	S	S
TJ151	female	68	2020/1/26	Sputum or throat swab	MSSA	ST398	CC398	t571	Absent	Absent	5	R	S	S	R	R	S	S	S	S	S	S	S	S	S	S
TJ152	male	83	2020/2/25	Sputum or throat swab	MSSA	ST398	CC398	t1451	Absent	Absent	5	S	S	S	R	R	I	S	S	S	S	S	S	S	S	S
TJ155	female	86	2020/4/5	Sputum or throat swab	MSSA	ST15	CC15	unknown	Absent	Absent	8	R	S	S	R	R	S	S	S	S	S	S	S	S	S	S
TJ156	female	66	2020/4/5	Pus or wound exudate	MSSA	ST6	CC5	t2467	Absent	Absent	8	R	S	S	S	S	S	S	S	S	S	S	S	S	S	S
TJ157	male	71	2020/4/15	Pus or wound exudate	MSSA	ST188	CC1	t189	Absent	Absent	8	R	S	S	R	R	S	S	S	S	S	S	S	S	S	S
TJ158	male	29	2020/5/2	Pus or wound exudate	MSSA	ST22	CC22	unknown	Present	Absent	5	R	S	S	R	R	S	S	S	S	S	S	S	S	S	S
TJ159	female	72	2020/5/7	Others	MSSA	ST398	CC398	t1580	Absent	Absent	5	S	S	S	S	S	S	S	S	S	S	S	S	S	S	S
TJ160	female	72	2020/5/5	Blood	MSSA	ST398	CC398	unknown	Absent	Absent	5	S	S	S	S	S	S	S	S	S	S	S	S	S	S	S
TJ161	male	62	2020/5/8	Urine	MRSA	ST398	CC398	t034	Absent	V(5C2)	5	R	R	R	R	R	S	S	S	S	S	S	S	S	S	S
TJ162	female	78	2020/5/13	Pus or wound exudate	MSSA	ST1281	CC1281	unknown	Absent	Absent	5	R	S	S	S	S	S	S	S	S	S	S	S	S	S	S
TJ163	male	24	2020/5/18	Pus or wound exudate	MRSA	ST398	CC398	t011	Absent	V(5C2)	5	R	R	R	R	R	S	S	S	S	S	S	S	S	S	S
TJ164	male	21	2020/6/24	Pus or wound exudate	MRSA	ST22	CC22	t309	Present	Absent	5	R	R	R	S	S	S	S	S	S	S	S	S	S	S	S
TJ165	male	24	2020/6/23	Others	MRSA	ST59	CC59	t437	Present	Vb(5C2&5)	8	R	R	R	R	R	S	R	S	S	S	S	S	S	S	S
TJ166	male	72	2020/6/27	Pus or wound exudate	MSSA	ST398	CC398	t571	Absent	Absent	5	R	S	S	S	S	S	S	S	S	S	S	S	S	S	S
TJ167	female	79	2020/7/1	Blood	MSSA	ST5	CC5	t002	Absent	Absent	5	R	S	S	R	R	R	S	I	S	S	R	S	S	S	S
TJ169	female	40	2020/7/18	Pus or wound exudate	MRSA	ST2114	CC5	t701	Absent	Absent	8	R	R	R	S	S	S	S	S	S	S	S	S	S	S	S
TJ170	female	65	2020/8/7	Blood	MRSA	ST15	CC15	unknown	Absent	Absent	8	R	R	R	S	S	S	S	S	S	S	S	S	S	S	S
TJ171	male	13	2020/8/16	Others	MSSA	ST188	CC1	t189	Absent	Absent	8	R	S	S	S	S	S	S	S	S	S	S	S	S	S	S
TJ172	male	58	2020/9/3	Pus or wound exudate	MSSA	ST1281	CC1281	t164	Absent	Absent	5	R	S	S	S	S	S	S	S	S	S	S	S	S	S	S
TJ173	female	89	2020/9/13	Pus or wound exudate	MRSA	ST1281	CC1281	t164	Absent	Absent	5	R	R	R	R	R	S	S	I	S	S	S	S	S	S	S
TJ174	male	36	2020/9/18	Pus or wound exudate	MRSA	ST398	CC398	t034	Absent	V(5C2)	5	R	R	R	R	R	S	S	S	S	S	S	S	S	S	S
TJ175	male	58	2020/9/16	Pus or wound exudate	MSSA	ST1281	CC1281	t164	Absent	Absent	5	R	S	S	S	S	S	S	S	S	S	S	S	S	S	S
TJ176	male	79	2020/9/19	Others	MSSA	ST7	CC7	t796	Absent	Absent	8	R	S	S	R	R	R	S	S	S	S	R	S	S	S	S
TJ177	male	79	2020/9/23	Others	MSSA	ST7	CC7	t796	Absent	Absent	8	R	S	S	R	R	R	S	S	S	S	R	S	S	S	S
TJ178	female	83	2020/10/2	Pus or wound exudate	MRSA	ST338	CC59	t437	Present	Vb(5C2&5)	unknown	R	R	R	R	R	S	R	S	S	S	S	S	S	S	S
TJ179	male	64	2020/10/4	Pus or wound exudate	MSSA	ST6	CC5	t701	Absent	Absent	8	R	S	S	S	S	S	S	S	S	S	S	S	S	S	S
TJ180	male	38	2020/10/8	Pus or wound exudate	MRSA	ST59	CC59	t437	Absent	IVa(2B)	8	R	R	R	S	S	S	S	S	S	S	S	S	S	S	S
TJ181	female	35	2020/10/8	Pus or wound exudate	MSSA	ST398	CC398	unknown	Present	Absent	5	R	S	S	R	R	S	S	S	S	S	S	S	S	S	S
TJ182	male	60	2020/10/10	Pus or wound exudate	MSSA	ST6732	Unknown	t14133	Absent	Absent	5	R	S	S	S	S	S	S	R	R	I	S	S	S	S	S
TJ183	female	83	2020/10/14	Pus or wound exudate	MRSA	ST338	CC59	t437	Present	Vb(5C2&5)	8	R	R	R	R	R	S	S	S	S	S	S	S	S	S	S
TJ184	male	67	2020/11/7	Blood	MRSA	ST5527	CC8	t030	Absent	III(3A)	8	R	R	R	S	S	R	R	R	R	R	S	R	S	S	S
TJ185	male	22	2020/12/4	Pus or wound exudate	MRSA	ST59	CC59	t1751	Present	IVa(2B)	8	R	R	R	R	R	S	R	I	S	S	S	S	S	S	S
TJ186	female	74	2020/12/6	Blood	MRSA	ST2114	CC5	t701	Absent	Absent	8	R	R	R	S	S	S	S	S	S	S	S	S	S	S	S
TJ187	male	29	2020/12/11	Pus or wound exudate	MRSA	ST1	CC1	t114	Absent	Absent	8	R	R	R	R	R	S	S	S	S	S	S	S	S	S	S
TJ188	male	25	2021/1/3	Pus or wound exudate	MSSA	ST22	CC22	t309	Present	Absent	5	R	S	S	S	S	S	S	S	S	S	S	S	S	S	S
TJ189	female	70	2021/1/4	Pus or wound exudate	MRSA	ST5529	CC5	t688	Absent	IVg(2B)	5	R	R	R	R	R	S	S	S	S	S	S	S	S	S	S
TJ190	male	89	2021/1/21	Sputum or throat swab	MSSA	ST752	Unknown	t4652	Absent	Absent	5	S	S	S	S	S	S	S	S	S	S	S	S	S	S	S
TJ191	male	21	2021/1/25	Pus or wound exudate	MSSA	ST188	CC1	t189	Absent	Absent	8	R	S	S	S	S	S	S	S	S	S	S	S	S	S	S
TJ192	male	56	2021/2/7	Blood	MSSA	ST1281	CC1281	t164	Absent	Absent	5	S	S	S	R	R	S	S	I	S	S	S	S	S	S	S
TJ193	female	88	2021/2/13	Others	MSSA	ST398	CC398	t034	Absent	Absent	5	R	S	S	S	S	S	S	S	S	S	S	S	S	S	S
TJ194	male	56	2021/2/23	Pus or wound exudate	MSSA	ST398	CC398	t571	Absent	Absent	5	R	S	S	S	S	S	S	S	S	S	S	S	S	S	S
TJ195	female	47	2021/3/6	Pus or wound exudate	MRSA	ST59	CC59	t437	Absent	IVa(2B)	8	R	R	R	R	R	S	S	I	S	S	S	S	S	S	S
TJ196	male	39	2021/3/24	Pus or wound exudate	MSSA	ST59	CC59	t8886	Absent	Absent	8	R	S	S	R	R	S	R	S	S	S	S	S	S	S	S
TJ197	male	85	2021/3/26	Urine	MSSA	ST5459	CC15	unknown	Absent	Absent	8	S	S	S	S	S	S	S	S	S	S	S	S	S	S	S
TJ198	female	74	2021/3/30	Others	MRSA	ST2114	CC5	t701	Absent	Absent	8	R	R	R	S	S	S	S	I	R	S	S	S	S	S	S
TJ199	male	55	2021/4/1	Pus or wound exudate	MSSA	ST22	CC22	t309	Absent	Absent	5	R	S	S	S	S	S	S	S	S	S	S	S	S	S	S
TJ200	male	39	2021/4/2	Pus or wound exudate	MRSA	ST59	CC59	t8886	Absent	Absent	8	R	R	R	R	R	S	R	S	S	S	S	S	S	S	S
TJ201	male	14	2021/4/3	Pus or wound exudate	MSSA	ST188	CC1	t189	Absent	Absent	8	R	S	S	S	S	S	S	S	S	S	S	S	S	S	S
TJ202	male	18	2021/4/7	Others	MSSA	ST398	CC398	t011	Present	Absent	5	R	S	S	S	S	S	S	S	S	S	S	S	S	S	S
TJ203	female	47	2021/4/7	Pus or wound exudate	MRSA	ST59	CC59	t437	Absent	IVa(2B)	8	R	R	R	R	R	S	S	I	I	S	S	S	S	S	S
TJ204	male	55	2021/4/8	Pus or wound exudate	MSSA	ST22	CC22	t309	Absent	Absent	5	R	S	S	S	S	S	S	S	S	S	S	S	S	S	S
TJ205	male	39	2021/4/13	Pus or wound exudate	MSSA	ST59	CC59	t8886	Absent	Absent	8	R	S	S	R	R	S	R	S	S	S	S	S	S	S	S
TJ206	male	33	2021/4/18	Pus or wound exudate	MRSA	ST6	CC5	t4562	Absent	Absent	8	R	R	R	S	S	S	S	S	S	S	S	S	S	S	S
TJ207	male	39	2021/4/26	Pus or wound exudate	MSSA	ST59	CC59	t8886	Absent	Absent	8	R	S	S	R	R	S	R	S	S	S	S	S	S	S	S
TJ208	female	62	2021/5/8	Others	MSSA	ST1	CC1	t127	Absent	Absent	8	S	S	S	R	R	S	S	S	S	S	R	S	S	S	S
TJ209	female	72	2021/5/18	Others	MRSA	ST25	CC25	t349	Absent	Absent	5	R	R	R	S	S	S	S	S	S	S	R	S	S	S	S
TJ210	male	97	2021/5/19	Sputum or throat swab	MSSA	ST22	CC22	t309	Absent	Absent	5	R	S	S	R	R	S	S	S	S	S	S	S	S	S	S
TJ211	male	47	2021/5/20	Others	MSSA	ST15	CC15	t385	Absent	Absent	8	R	S	S	R	R	S	S	S	S	S	S	S	S	S	S
TJ212	female	63	2021/5/24	Others	MSSA	ST188	CC1	t189	Absent	Absent	8	R	S	S	S	S	S	S	S	S	S	S	S	S	S	S
TJ213	male	68	2021/5/26	Others	MSSA	ST6	CC5	t701	Absent	Absent	8	R	S	S	S	S	S	S	S	S	S	S	S	S	S	S
TJ214	male	91	2021/5/28	Sputum or throat swab	MSSA	ST398	CC398	t034	Absent	Absent	5	R	S	S	R	R	S	S	S	S	S	S	S	S	S	S

Table S2. Primers and sizes of amplicons used for detection of virulence factor genes and capsule typing.

Primers	Sequence(5'-3')	Size (bp) of PCR product	Reference
Cap5-k1	GTCAAAGATTATGTGATGCTACTGAG	361	(1)
Cap5-k2	ACTTCGAATATAAACTTGAATCAATGTTATACAG		
Cap8-k1	GCCTTATGTTAGGTGATAAACC	173	(1)
Cap8-k2	GGAAAAACACTATCATAGCAGG		
Hla-F	CTGATTACTATCCAAGAAATTCGATTG	209	(2)
Hla-R	CTTTCCAGCCTACTTTTTTATCAGT		
Hlb-F	GTGCACTTACTGACAATAGTGC	309	(2)
Hlb-R	GTTGATGAGTAGCTACCTTCAGT		
HlgB-F	ATGAAAATGAATAAATTAGTCAAATCATCCGTTGC	792	(3)
HlgB-R	TTACTGTAATTTTAGATTTTTTAGCGCCATCTTG		
HlgC-F	GCTTAAAAATAAAATATTAACTACAACTTTATCTGTGAGC	720	(3)
HlgC-R	TCAATTCTGTCCTTTCACCTTGATTTCATGAG		
IcaC-F	ATGGGACGGATTCCATGAAAAAGA	1100	(4)
IcaC-R	TAATAAGCATTAATGTTCAATT		
Coa-F	ATA GAG ATG CTG GTA CAG G	630	(5)
Coa-R	GCT TCC GAT TGT TCG ATG C		
Nuc-F	GGTGAAACCGAATACGCCTGTA	197	(5)
Nuc-R	CTCTAGCAAGTCCCTTTTCCACTAA		

Table S3. Molecular characteristics of *S. aureus* isolates collected in this study

**CC (no.)**	**MLST (no.)**	**ST-*spa* (no.)**	**MSSA (no.)**	**MRSA (no.)**	**SCC*mec* (no.)**
CC59 (36)	ST59 (32)	ST59-t437 (19)	4	15	IVa(2B) (12), Vb(5C2&5) (3)
		ST59-t8886 (4)	3	1	None (1)
		ST59-t163 (3)	2	1	IVa(2B) (1)
		ST59-unknown (2)		2	IVa(2B) (1), Vb(5C2&5) (1)
		ST59-t441 (2)		2	IVa(2B) (2)
		ST59-t3736 (1)	1		
		ST59-t1751 (1)		1	IVa(2B) (1)
	ST338 (4)	ST338-t437 (4)	1	3	Vb(5C2&5) (3)
CC398 (30)	ST398 (30)	ST398-t034 (8)	6	2	V(5C2) (2)
		ST398-unknown (8)	8		
		ST398-t571 (5)	5		
		ST398-t1255 (2)	1	1	none (1)
		ST398-t011 (2)	1	1	V(5C2) (1)
		ST398-t11729 (1)		1	none (1)
		ST398-t1451 (1)	1		
		ST398-t1580 (1)	1		
		ST398-t4562 (1)	1		
		ST398-t588 (1)	1		
CC1 (25)	ST188 (13)	ST188-t189 (13)	12	1	none (1)
	ST1 (12)	ST1-t127 (8)	8		
		ST1-t114 (2)		2	IVg(2B) (1), none (1)
		ST1-t286 (1)	1		
		ST1-t948 (1)	1		
CC5 (24)	ST5 (9)	ST5-t002 (4)	4		
		ST5-t2958 (1)	1		
		ST5-t5353 (1)	1		
		ST5-t653 (1)	1		
		ST5-t688 (1)	1		
		ST5-unknown (1)		1	none (1)
	ST6 (8)	ST6-t701 (4)	4		
		ST6-t304 (2)		2	IVa(2B) (2)
		ST6-t2467 (1)	1		
		ST6-t4562 (1)		1	none (1)
	ST2114 (3)	ST2114-t701 (3)		3	none (3)
	ST3687 (1)	ST3687-t045 (1)	1		

	ST5529 (1)	ST5529-t688 (1)		1	IVg(2B) (1)
	ST965 (1)	ST965-t062 (1)		1	IVc(2B) (1)
	ST95 0(1)	ST950-t895 (1)	1		
CC8 (23)	ST5527 (13)	ST5527-t030 (13)		12	III(3A) (12)
		ST5527-unknown (1)		1	III(3A) (1)
	ST72 (5)	ST72-t148 (2)	2		
		ST72-unknown (1)	1		
		ST72-t664 (1)		1	IVc(2B) (1)
		ST72-t324 (1)		1	IVc(2B) (1)
	ST8 (2)	ST8-t9101 (2)	2		
	ST630 (2)	ST630-t11041 (1)	1		
		ST630-t377 (1)		1	none (1)
	ST1821 (1)	ST1821-t377 (1)	1		
CC22 (19)	ST22 (19)	ST22-t309 (13)	12	1	none (1)
		ST22-unknown (2)	2		
		ST22-t15234 (1)	1		
		ST22-t16061 (1)	1		
		ST22-t3668 (1)	1		
		ST22-t005 (1)		1	none (1)
ST25 (11)	ST25 (11)	ST25-t078 (4)	2	2	none (2)
		ST25-unknown (1)		1	IVa(2B) (1)
		ST25-t081 (1)	1		
		ST25-t1102 (1)	1		
		ST25-t1701 (1)	1		
		ST25-t18617 (1)	1		
		ST25-t2992 (1)	1		
		ST25-t349 (1)		1	none (1)
CC15 (8)	ST15 (7)	ST15-unknown (2)	1	1	none (1)
		ST15-t084 (2)	1	1	none (1)
		ST15-t11579 (1)	1		
		ST15-t2325 (1)	1		
		ST15-t385 (1)	1		
	ST5459 (1)	ST5459-unknown (1)	1		
ST1281 (6)	ST1281 (6)	ST1281-t164 (5)	4	1	none (1)
		ST1281-unknown (1)	1		
CC7 (5)	ST7 (5)	ST7-1867 (1)	1		
		ST7-t18004 (1)	1		
		ST7-t796 (1)	1		

		ST7-t797 (1)	1		
		ST7-t798 (1)	1		
CC2 (2)	ST88 (2)	ST88-t3155 (1)	1		
		ST88-unknown (1)		1	none (1)
CC30 (2)	ST30 (2)	ST30-t021 (2)	2		
CC121 (1)	ST121 (1)	ST121-t2019 (1)	1		
CC9 (1)	ST9 (1)	ST9-t899 (1)		1	XII(9C2) (1)
CC6310 (1)	ST6310 (1)	ST6310-t796 (1)	1		
CC6732 (1)	ST6732 (1)	ST6732-t14133 (1)	1		
CC752 (1)	ST752 (1)	ST752-t4562 (1)	1		
CC944 (1)	ST944 (1)	ST944-t616 (1)	1		
STNC (4)	STNC (4)	STNC-unknown (1)		1	IVa(2B) (1)
		STNC-t2883 (1)	1		
		STNC-t377 (1)	1		
		STNC-t954 (1)	1		

References

1. Verdier I, Durand G, Bes M, Taylor KL, Lina G, Vandenesch F, et al. 2007. Identification of the capsular polysaccharides in *Staphylococcus aureus* clinical isolates by PCR and agglutination tests. J Clin Microbiol. 2007; 45(3):725–729.
2. Jung HR, Lee YJ. Characterization of virulence factors in enterotoxin-producing *Staphylococcus aureus* from bulk tank milk. Animals (Basel). 2022; 12(3):301.
3. Lin T, Li Q, Jin D, Liu W, Tang C, Zhang X. Investigation of virulence genes of *Staphylococcus aureus* isolated from sterile body fluid samples and their correlation with clinical symptoms and outcomes. Can J Infect Dis Med Microbiol . 2021; 2021:5354747.
4. Mohammadi MC, Anzabi Y, Shayegh J. Comparison of the frequency of biofilm-forming genes (*icaABCD*) in methicillin-resistant *S. aureus* strains isolated from human and livestock. Arch Razi Inst. 2021; 76(6):1655–1663.
5. Kuroda M, Ohta T, Uchiyama I, Baba T, Yuzawa H, Kobayashi I, et al. Whole genome sequencing of meticillin-resistant *Staphylococcus aureus*. Lancet. 2001; 357(9264):1225–1240.
